# A promoter-proximal transcript targeted by genetic polymorphism controls E-cadherin silencing in human cancers

**DOI:** 10.1038/ncomms15622

**Published:** 2017-05-30

**Authors:** Giuseppina Pisignano, Sara Napoli, Marco Magistri, Sarah N. Mapelli, Chiara Pastori, Stefano Di Marco, Gianluca Civenni, Domenico Albino, Claudia Enriquez, Sara Allegrini, Abhishek Mitra, Gioacchino D'Ambrosio, Maurizia Mello-Grand, Giovanna Chiorino, Ramon Garcia-Escudero, Gabriele Varani, Giuseppina M. Carbone, Carlo V. Catapano

**Affiliations:** 1 Tumor Biology and Experimental Therapeutics Program, Institute of Oncology Research (IOR), and Oncology Institute of Southern Switzerland (IOSI), Bellinzona 6500, Switzerland; 2 IRCCS Multimedica, Milan 20099, Italy; 3 Laboratory of Cancer Genomics, Fondo Edo Tempia, Biella 13900, Italy; 4 Molecular Oncology Unit, CIEMAT and Centro de Investigación Biomédica en Red de Cáncer (CIBERONC), Madrid 28040, Spain; 5 Department of Chemistry, University of Washington, Seattle, Washington 98195-1700, USA; 6 Department of Oncology, Faculty of Biology and Medicine, University of Lausanne, Lausanne 1066, Switzerland

## Abstract

Long noncoding RNAs are emerging players in the epigenetic machinery with key roles in development and diseases. Here we uncover a complex network comprising a promoter-associated noncoding RNA (paRNA), microRNA and epigenetic regulators that controls transcription of the tumour suppressor E-cadherin in epithelial cancers. E-cadherin silencing relies on the formation of a complex between the paRNA and microRNA-guided Argonaute 1 that, together, recruit SUV39H1 and induce repressive chromatin modifications in the gene promoter. A single nucleotide polymorphism (rs16260) linked to increased cancer risk alters the secondary structure of the paRNA, with the risk allele facilitating the assembly of the microRNA-guided Argonaute 1 complex and gene silencing. Collectively, these data demonstrate the role of a paRNA in E-cadherin regulation and the impact of a noncoding genetic variant on its function. Deregulation of paRNA-based epigenetic networks may contribute to cancer and other diseases making them promising targets for drug discovery.

## Abstract (web summary)

Promoter-proximal transcripts have been proposed to act as *cis*-acting elements regulating transcription. Here, the authors provide evidence that a promoter-proximal RNA, in combination with other epigenetic regulators, controls transcription of E-cadherin in epithelial cancers.

The human genome is pervasively transcribed generating a large number of non-coding transcripts, called non-coding RNAs (ncRNAs), most with yet undefined function^1^,^2^. Long intergenic ncRNAs (lincRNAs) and natural antisense transcripts (NATs) have been shown to act as guides for recruitment of epigenetic effectors to specific genomic sites and have been implicated in transcriptional reprogramming during development and disease^1^,^3^,^4^. High-throughput RNA sequencing efforts have revealed that long ncRNAs are also transcribed in promoter-proximal regions of most annotated genes^5^,^6^,^7^,^8^. Promoter–proximal transcripts (hereafter referred as promoter-associated RNAs, paRNAs) might act as *cis*-acting elements in transcriptional regulation of neighbouring genes but their molecular and functional characterization is still largely lacking^9^. An intriguing question is whether genetic variants, like single-nucleotide polymorphisms (SNPs), in promoter regions affect the function of promoter-proximal transcripts. Several disease-associated SNPs map to noncoding regions, including promoters^10^,^11^,^12^,^13^, and may underlie allele-specific differences in the local epigenetic landscape by altering DNA elements, like transcription factor-binding sites^14^,^15^,^16^. However, a clear mechanistic link between noncoding SNPs and transcriptional regulation is in many cases missing^17^.

In this study, we examine the transcriptional landscape within the promoter–proximal region of the gene encoding E-cadherin (CDH1), an important epithelial cell differentiation and tumour suppressor protein^18^. CDH1 is epigenetically silenced in many epithelial cancers and this event is associated with the epithelial-to-mesenchymal transition (EMT) and the acquisition of cancer stem-like cell (CSC) properties, key features in tumour progression^18^. We find that silencing of CDH1 involves the formation of a microRNA (miRNA)-guided Argonaute 1 (AGO1) complex on a sense promoter-proximal transcript with consequent recruitment of SUV39H1 and repressive chromatin modifications. We provide evidence that a cancer-associated SNP influences this paRNA-based regulatory network by modifying the secondary structure of the sense transcript within the miRNA/AGO1 binding region, thereby increasing the accessibility of the miRNA/AGO1 complex to the paRNA and the CDH1 promoter. Altogether, we show the involvement of paRNAs in the transcriptional regulation of a key tumour suppressor gene and how the impact of a functional noncoding genetic variant on epigenetic gene silencing and tumour progression is mediated by the effect on the secondary structure of a promoter-proximal transcript. Our work suggests that deregulation of paRNA-based transcriptional mechanisms could contribute to epigenetic silencing of tumour suppressor genes and disease progression in cancer patients.

## Results

### Promoter-proximal transcripts at the *CDH1* gene locus

**Figure 1:**
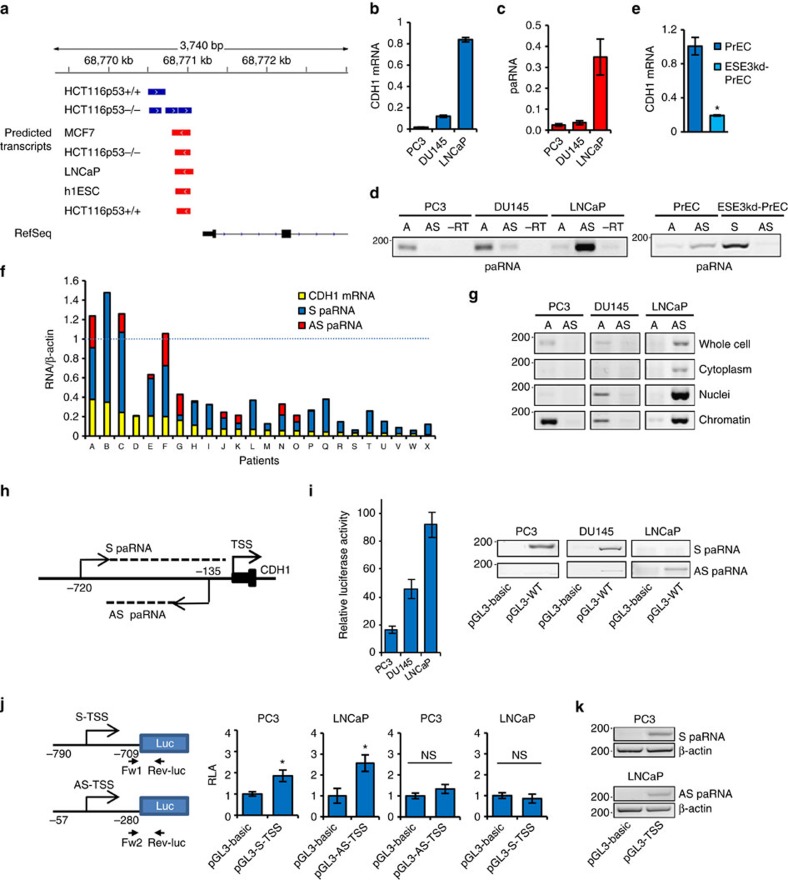
Promoter-associated transcripts at the *CDH1* gene locus. (**a**) Predicted promoter-proximal transcripts with sense (blue) and antisense (red) orientation relative to the *CDH1* gene from GRO-Seq data in multiple cell lines. (**b**,**c**) Level of CDH1 mRNA (**b**) and paRNA (**c**) in prostate cancer cell lines determined by qRT-PCR. (**d**) S- and AS-paRNAs in prostate cancer cell lines determined by strand-specific RT–PCR. −RT, control reaction without RT step. (**e**) Levels of CDH1 mRNA and paRNAs in normal PrECs and tumorigenic ESE3kd-PrECs determined by qRT–PCR (top) and strand-specific RT–PCR (bottom). (**f**) S-paRNA (blue bars), AS-paRNA (red bars) and CDH1 mRNA (yellow bars) determined by qRT-PCR in human prostate tumours. Dotted line, CDH1 mRNA level in normal PrECs shown as reference. (**g**) Intracellular distribution of S- and AS-paRNA in prostate cancer cell lines. (**h**) S and AS transcript initiation sites in the CDH1 promoter determined by 5′-RACE (see Supplementary Fig. 2d). (**i**) Luciferase reporter activity (left) and promoter–proximal transcripts (right) generated from the full length CDH1 promoter construct in prostate cancer cell lines. (**j**) Schematic representation of S-TSS and AS-TSS promoter reporter constructs and luciferase activity in PC3 and LNCaP cells. (**k**) S and AS transcripts generated from S-TSS and AS-TSS constructs in PC3 and LNCaP cells determined by strand-specific RT–PCR. Positions of forward and reverse primers for RT–PCR are indicated in **j**. Data are mean±s.d. of three independent determinations. Student's *t*-test was used for *P* value assessment. *P<0.05.

We applied transcript prediction tools in the Hypergeometric Optimization of Motif EnRichment (HOMER) suite for Motif Discovery and next-generation sequencing analysis^19^ to a compendium of GRO-Seq data sets^20^,^21^,^22^,^23^ from human cell lines to examine the transcriptional landscape at the CDH1 promoter. Antisense (AS) and sense (S) oriented transcripts were predicted with high reproducibility in different cell lines (Fig. 1a). All the cell lines exhibited promoter-associated transcripts in the AS orientation relative to the gene transcription start site (TSS), consistent with the presence of bidirectional transcripts at many active promoters. Along with the AS transcripts, we detected also low levels of S-oriented transcripts in the CDH1 promoter in HCT116 cells (Supplementary Fig. 1a). Interestingly, HCT116 cells had the lowest CDH1 expression in this cell line panel (Supplementary Fig. 1b).

To verify these predictions, we examined the presence of promoter-proximal transcripts in a panel of prostate cancer cell lines with differential CDH1 expression (Fig. 1b). Using quantitative reverse transcription PCR (qRT–PCR), we detected paRNAs with cumulative levels proportional to CDH1 expression (Fig. 1c). Strand-specific RT–PCR showed that LNCaP cells, with the highest CDH1 expression, exhibit prevalently AS-paRNA (Fig. 1d). Conversely, PC3 and DU145 cells with low CDH1 expression had a greater amount of S-paRNA relative to AS-paRNA.

We further evaluated the relationship between paRNA and CDH1 expression in normal and tumorigenic prostate epithelial cells (PrECs). We showed previously that knockdown of the transcription factor ESE3/EHF (EHF) in PrECs induces a tumorigenic phenotype along with EMT, CSC-like properties and CDH1 repression^24^. We find that repression of E-cadherin in the tumorigenic EHFkd-PrECs is associated with decreased AS-paRNA and increased S-paRNA expression (Fig. 1e). Notably, human prostate tumours, which exhibit significantly lower E-cadherin expression compared to normal PrECs, have almost invariably a higher level of S-paRNA than AS-paRNA (Fig. 1f). Thus, both in cell lines and human samples, the relative abundance of S-paRNA and AS-paRNA characterizes distinct states of the *CDH1* gene and the prevalence of S-paRNA is associated with attenuated CDH1 transcription.

### 3′-termini of CDH1 promoter-associated transcripts

We examined the cellular distribution of paRNA by performing strand-specific RT–PCR on RNA extracted from PC3, DU145 and LNCaP cells after subcellular fractionation. S and AS-paRNAs are mainly nuclear and chromatin-bound (Fig. 1g), consistent with an epigenetic function. Interestingly, the chromatin fraction contains exclusively S-paRNA in PC3 and DU145 cells and AS-paRNA in LNCaP cells. To establish whether the paRNA are poly-adenylated, we compared the level of the transcripts detected by retro-transcribing either total or poly(A)+ RNAs. Compared to β-actin mRNA, the majority of promoter transcripts does not have poly(A) tails, and only a minor portion is poly-adenylated in PC3 (∼40%) and LNCaP (∼20%) cells (Supplementary Fig. 2a).

We next performed 3′ rapid amplification of cDNA ends (3′RACE) after adding poly(A) tails to total RNA extracted from PC3 and LNCaP cells. A single prominent AS transcript is detected in LNCaP cells with a unique termination site −456 bp from the CDH1 TSS (Supplementary Fig. 2b). Conversely, 3′RACE for the S transcript reveals multiple transcripts in PC3 cells with termination sites extending up to −54 and −10 bp upstream the CDH1 TSS. These findings indicate that the S-oriented transcripts are independent and not extended variants of CDH1 mRNA.

To verify whether the heterogeneity of the 3′-ends of the S transcripts is due to progressive degradation, we knocked down Rrp44, a core subunit of the exosome^6^. The level of S-paRNA in PC3 cells increases after Rrp44 knockdown indicating that degradation by the exosome contributes to the low steady-state level and the progressive shortening of the sense transcript (Supplementary Fig. 2c). Interestingly, 3′RACE performed on the soluble nuclear RNA fraction shows an increasing number of shorter transcripts ending further upstream of the TSS (−150 to −220 bp), suggesting that transcripts detached from chromatin are more susceptible to degradation by the exosome.

### Promoter-associated transcript initiation sites

The consistent location of the 5′ termini of the transcripts predicted by GRO-Seq data analysis in multiple cell lines suggests the presence of specific initiation events in the CDH1 promoter (Fig. 1a). We performed 5′ rapid amplification of cDNA ends (5′RACE) to determine the 5′-termini of the S and AS transcripts in PC3 and LNCaP cells. In both cell lines, a single transcript is detected in either S or AS orientation. Sequencing of the 5′RACE products reveals that the 5′-end of the S transcript is located −720 bp from the CDH1 TSS, while the 5′ end of the AS transcript maps to −135 bp from the CDH1 TSS (Supplementary Fig. 2d). Both S and AS transcript initiation sites agree with the 5′-termini predicted from GRO-seq data (Fig. 1h). Furthermore, the position of the AS-paRNA initiation site (AS-TSS) is within the range (≤250 bp) reported by others for promoter–proximal AS transcripts^25^,^26^.

These results support the presence of precise and independent S and AS-transcript initiation sites in the CDH1 promoter. To test this hypothesis, we turned to a CDH1 promoter reporter construct and found that it reproduces the activity of the native promoter along with the distinctive pattern of noncoding transcripts in each cell line (Fig. 1i). Next, we cloned the fragments encompassing the putative sense (S-TSS, −790 to −709) and antisense (AS-TSS, −57 to −280) initiation sites in a promoter-less luciferase reporter (Fig. 1j, left panels). The AS-TSS and S-TSS reporters exhibit promoter activity in LNCaP and PC3 cells, respectively (Fig. 1j, middle panels). Conversely, constructs in opposite orientation (S-TSS and AS-TSS) are not active in the same cells (Fig. 1j, right panels). These findings were confirmed by detecting with RT-PCR the chimeric transcripts produced by AS-TSS and S-TSS reporters in LNCaP and PC3 cells (Fig. 1k). Thus, the cloned promoter fragments were able to drive transcription directionally and independently of the gene TSS in the proper cell context. Control experiments demonstrated that the transcripts derived from the reporter constructs (Supplementary Fig. 2e). Furthermore, deletion of the upstream region (−790 to −373) containing the S-TSS abolished transcription of S-paRNA in PC3 cells but allowed the synthesis of AS-paRNA in LNCaP cells (Supplementary Fig. 2f), consistent with independent regulation of the S and AS-transcript initiation sites in the promoter-proximal regions of the *CDH1* gene.

### Sense promoter-associated RNA silences CDH1

**Figure 2:**
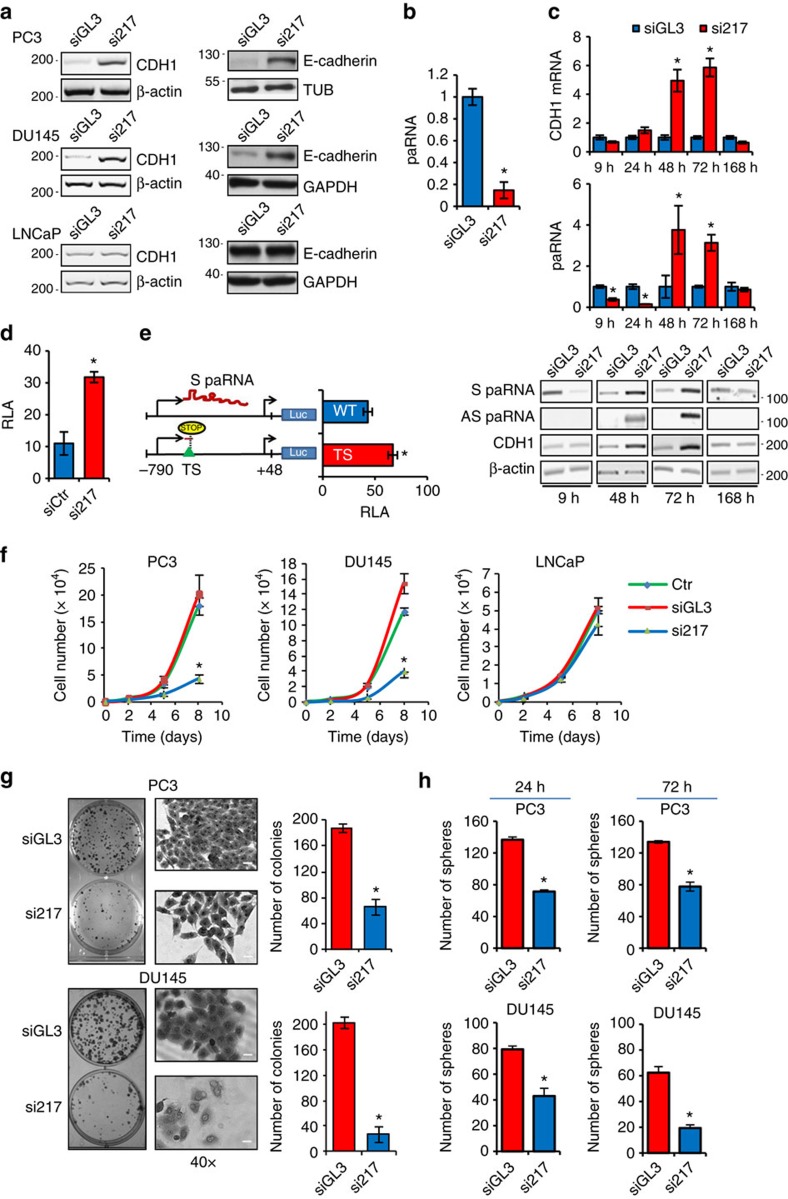
Promoter-associated RNAs control CDH1 expression. (**a**) Expression of CDH1 mRNA (left) and E-cadherin protein (right) 72 h after transfection with si217 or control (siGL3) siRNA. (**b**) paRNA levels in PC3 cells 24 h after transfection with siGL3 or si217. (**c**) CDH1 mRNA and paRNAs in PC3 cells after transfection with si217 or siGL3. Top and middle panels, samples analysed by qRT–PCR (CDH1 mRNA and paRNA); lower panel, samples analysed by RT–PCR (CDH1 and β-actin mRNA) or strand-specific RT-PCR (S and AS paRNA). (**d**) Luciferase activity in PC3 cells transfected with CDH1 promoter reporter and either si217 or siGL3. (**e**) Luciferase activity in PC3 cells transfected with CDH1 promoter reporter with either wild-type (WT) sequence or the insertion of a termination site (TS) constituted by the SV40 polyA cassette blocking synthesis of full length S-paRNA (Supplementary Fig. 3f). Left, schematic representation of the WT and TS reporter constructs. (**f**) Proliferation of prostate cancer cells transfected with siGL3, si217 or mock transfected (Ctr). (**g**) Colony formation by PC3 (top panels) and DU145 (bottom panels) cells transfected with si217 or siGL3. Scale bar, 20 μm. (**h**) Tumour sphere formation by PC3 and DU145 cells transfected with si217 or siGL3 and plated after 24 or 72 h in stem cell-selective conditions. Data are mean±s.d. of three independent determinations. Student's *t*-test was used for *P* value assessment. *P<0.05.

The differential expression of S and AS-paRNA in CDH1 high (CDH1^H^) and low (CDH1^L^) expressing cells suggests distinct roles for these noncoding transcripts in gene regulation. The presence of AS-paRNA might have a permissive role in transcriptional initiation and elongation^23^,^27^. Conversely, the prevalence of S-paRNA in CDH1^L^ cells suggests that this transcript could contribute to gene silencing. To verify this hypothesis, we used small interfering RNAs (siRNAs) to target the S-paRNA and examine the effect on CDH1 transcription. Promoter-directed siRNAs have been shown to induce transcriptional repression or activation by diverse mechanisms depending on promoter architecture and transcriptional state of genes^28^,^29^,^30^. Using Sfold we selected siRNAs that target accessible regions in the S-paRNA and exhibit greater strand-specificity, based on differential stability of siRNA duplex ends (DSSE >1 kcal mol^−1^). Si217 ranked at the top, based on these criteria, among siRNAs targeting the S-paRNA. Transfection of si217 reactivates CDH1 expression in PC3 and DU145 cells, but had no effect in LNCaP cells (Fig. 2a). Additional siRNAs (si304 and si63) directed to distinct non-overlapping sites also reactivate CDH1 transcription, although less effectively than si217 (Supplementary Fig. 3a,b). Re-expression of CDH1 involves AGO2 (Supplementary Fig. 3c) and was associated with depletion of S-paRNA (Fig. 2b and Supplementary Fig. 3d), suggesting that siRNA-loaded AGO2 interacts with and degrades the S-paRNA to activate CDH1 transcription. To confirm the strand-specificity of si217 we examined the effect in LNCaP cells, which express almost exclusively AS-paRNA. CDH1 mRNA and paRNA levels were not affected by the S-paRNA targeting si217 (Supplementary Fig. 3e).

To assess the dynamic relationship between paRNAs and CDH1 transcription, we monitored both mRNA and paRNA levels at different times after siRNA transfection. Depletion of S-paRNA is followed by increased CDH1 mRNA expression at 48–72 h (Fig. 2c). This occurs concomitantly with an increase of total paRNAs and, specifically, AS-paRNA as documented by qRT–PCR and strand-specific RT–PCR (Fig. 2c, middle and bottom panels), leading to an inversion of the AS/S-paRNA ratio. At the latest time point, reduced CDH1 mRNA levels coincide with decreased paRNA and the disappearance of AS-paRNA. Thus, the level and the balance between S and AS-paRNA dictate the status of the CDH1 promoter. Furthermore, transient depletion of S-paRNA triggers transcriptional reactivation in CDH1^L^ cells.

To gain additional insights on the mode of action of the S-paRNA, we turned to the CDH1 promoter reporter construct. We find that si217 increases promoter reporter activity, as observed with the native promoter (Fig. 2d). Moreover, insertion of a transcription termination site (TS) downstream of the S-TSS prevents synthesis of full length S-paRNA (Supplementary Fig. 3f) and increases promoter reporter activity (Fig. 2e). Conversely, ectopic expression of S and AS-paRNA has no effect on promoter reporter activity and CDH1 mRNA, indicating that the promoter-proximal transcripts do not have *trans-*acting effects (Supplementary Fig. 3g). Altogether, these results support the notion that S-paRNA acts locally at the CDH1 promoter and regulates E-cadherin transcription in *cis*.

### Sense promoter-associated RNA affects cancer cell phenotype

Our results show the S-paRNA has a central role in maintaining the silenced state of CDH1 in prostate cancer cell lines: depletion of the S paRNA re-activates E-cadherin expression in CDH1^L^ cancer cells. Given the importance of E-cadherin silencing for epithelial cancers^18^, we addressed whether preventing S-paRNA mediated repression reverts the tumorigenic phenotype. Restoring E-cadherin expression by various means is known to induce growth arrest and impair tumorigenicity of cancer cells^31^,^32^,^33^. We find that transfection of si217 inhibits proliferation of PC3 and DU145 cells, but has no effect in CDH1^H^ LNCaP cells (Fig. 2f). si63 and si304 had similar effects on proliferation of PC3 cells (Supplementary Fig. 3h). Clonogenicity of CDH1^L^ cells is also severely impaired by si217, giving rise to small clusters of scattered cells compared to the large colonies formed by control cells (Fig. 2g). Transfection with si217 also reduces the ability of PC3 and DU145 cells to form tumour spheres, a feature of CSCs (Fig. 2h). Reduced tumour sphere formation *in vitro* correlates with impaired tumour-initiating and stem-like capability *in vivo*^24^,^34^. Thus, targeting the S-paRNA reverts the tumorigenic and stem-like phenotype concomitant with reactivation of CDH1 expression. Altogether, these results confirm the pivotal role of the S-paRNA in sustaining E-cadherin silencing and the malignant phenotype.

### Sense promoter-associated RNA recruits AGO1 and SUV39H1

**Figure 3:**
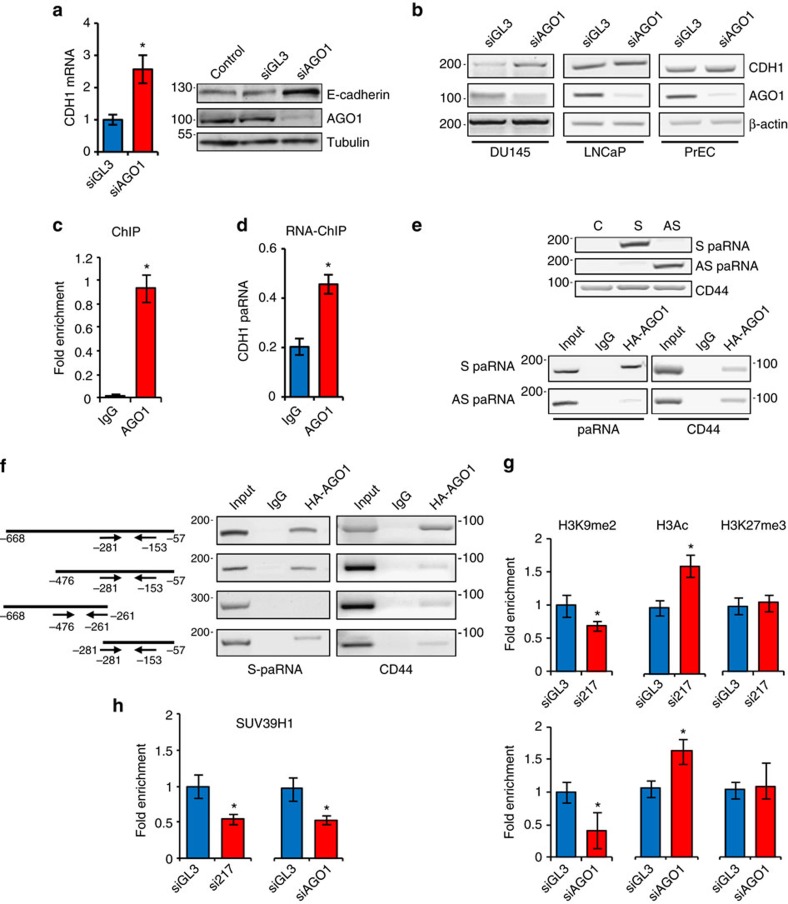
Argonaute 1 and promoter-associated RNA coordinate CDH1 gene silencing. (**a**) CDH1 mRNA (left) and protein levels (right) in PC3 cells transfected for 72 h with siAGO1 or siGL3. (**b**) CDH1 mRNA in DU145, LNCaP and PrECs transfected with siAGO1 or siGL3 and collected after 72 h. (**c**) AGO1 binding to the CDH1 promoter determined by ChIP (primers F-281/R-139). (**d**) Binding of AGO1 to S-paRNA determined by RNA-ChIP. (**e**) Expression of S and AS-paRNA (top) and binding to AGO1 (bottom) determined by RIP in PC3 cells transfected with paRNA and AGO1 expression vectors. CD44 was used as positive control for AGO1-bound mRNA. (**f**) Binding of truncated S-paRNA transcripts to AGO1 determined by RIP. Arrows, forward and reverse primer sets for each transcript. (**g**) Histone mark modifications at the CDH1 promoter in PC3 cells transfected with si217 (top) or siAGO1 (bottom) and control siGL3. (**h**) Binding of SUV39H1 to the CDH1 promoter in PC3 cells transfected with si217 (left) or siAGO1 (right) and siGL3. Data are mean±s.d. of three independent determinations. Student's *t*-test was used for *P* value assessment. *P<0.05.

To uncover the mechanism by which S-paRNA modulates CDH1 transcription, we sought to identify additional components of this regulatory network. We focused on AGO proteins, which are implicated in transcriptional gene regulation^35^,^36^. We find that knockdown of AGO1 increases CDH1 expression (Fig. 3a), while depletion of other AGOs has no effect (Supplementary Fig. 4a,b). AGO1 knockdown also increases CDH1 expression in DU145 cells, but no changes are observed in LNCaP and normal PrECs (Fig. 3b). We hypothesized that AGO1 could target the CDH1 promoter by interacting with the S-paRNA. We found AGO1 in the nucleus (Supplementary Fig. 4c) and bound to the CDH1 promoter in PC3 cells in the region overlapping the S-paRNA (Fig. 3c). Furthermore, RNA-chromatin immunoprecipitation (RNA-ChIP) show that chromatin-associated AGO1 binds to S-paRNA, a result consistent with the hypothesis that S-paRNA guides AGO1 to the promoter (Fig. 3d). This finding was confirmed by RNA-immunoprecipitation (RIP) in HA-AGO1 expressing PC3 cells (Supplementary Fig. 4d). Binding of AGO1 to S-paRNA is also observed in DU145 cells, but not in LNCaP cells (Supplementary Fig. 4e), which do not express the S-transcript. Thus, the interaction of AGO1 with S-paRNA occurs in CDH1^L^ cells concomitantly with silencing of the gene. Consistent with a repressive function, expression of HA-AGO1 reduces the activity of the CDH1 promoter reporter in PC3 cells, whereas it is ineffective in LNCaP cells lacking S-paRNA (Supplementary Fig. 4f). Furthermore, blocking synthesis of S-paRNA in the promoter reporter by the TS insertion reduces binding of AGO1, confirming that the S-transcript is required for AGO1 interaction with the promoter (Supplementary Fig. 4g).

We examined further the interaction of AGO1 with S-paRNA using RIP and paRNA expression constructs. AGO1 binds exclusively to the S and not to the AS-transcript, indicating that the binding is strand and sequence specific (Fig. 3e). Importantly, this experiment reveals that AGO1 binds directly to the S-paRNA independently of promoter DNA. Analysis of S-paRNA deletion mutants identify the 3′ portion of the transcript as the minimal region required for AGO1 binding, further arguing for selective sequence-specific interaction (Fig. 3f). Interestingly, AGO1 is unable to bind ectopically expressed S- and AS-paRNA in LNCaP cells (Supplementary Fig. 4h), indicating that formation of the S-paRNA-AGO1 complex is cell-context dependent and requires additional cell-specific elements.

To determine whether AGO1 and S-paRNA influence the chromatin state at the CDH1 promoter, we examined the distribution of activating (H3Ac) and repressive (H3K9me, H3K27me) histone marks after their knockdown in PC3 cells. Consistent with a switch to an active state, knockdown of S-paRNA and AGO1 increases H3Ac and decreases H3K9me at the CDH1 promoter (Fig. 3g). Notably, the restoration of an active chromatin environment for the CDH1 promoter after AGO1 knockdown is also associated with increased AS-paRNA, as seen after S-paRNA depletion (Supplementary Fig. 4i).

The selective decline of H3K9me, without changes in H3K27me, after S-paRNA and AGO1 knockdown suggests the specific involvement of a H3K9 histone methyltransferase (HMT), like SUV39H1 or G9a. We showed previously that knockdown of SUV39H1 reactivates CDH1 expression whereas G9a knockdown had no effect^37^. Consistent with this past result, knockdown of SUV39H1 reduces the levels of H3K9me at the CDH1 promoter and increases CDH1 expression in PC3 cells (Supplementary Fig. 4j–l). Treatment with chaetocin, a selective SUV39H1 inhibitor, similarly reactivates CDH1 transcription (Supplementary Fig. 4m). Consistent with a link with paRNA, knockdown of either S-paRNA or AGO1 reduces the amount of SUV39H1 bound to the CDH1 promoter (Fig. 3h). Furthermore, we found that SUV39H1 binds to ectopically expressed S-paRNA in the presence of AGO1 (Supplementary Fig. 4n). These findings suggest that AGO1 promotes the interaction of SUV39H1 with S-paRNA and facilitates its subsequent recruitment to the CDH1 promoter. In turn, SUV39H1 recruited by the S-paRNA-AGO1 complex catalyses H3K9me and silencing of the gene.

### A microRNA guides AGO1 to sense promoter-associated RNA

**Figure 4:**
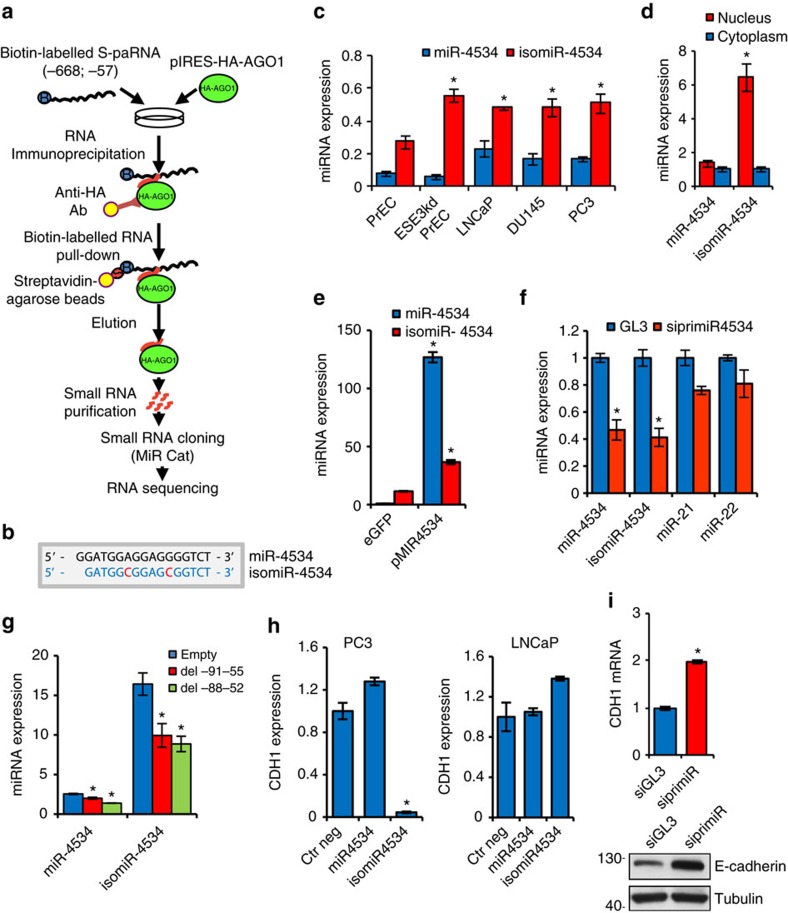
IsomiR-4534 binds to Argonaute 1 and controls CDH1 expression. (**a**) Protocol for miRNA identification by RIP, biotin-labelled RNA pull-down and small RNA sequencing. (**b**) Sequence of miR-4534 and isomiR-4534. Red, nucleotide changed in isomiR-4534. (**c**) miR-4534 and isomiR-4534 expression in prostate normal (PrECs), tumorigenic (ESE3kd-PrECs) and cancer cell lines (LNCaP, DU145 and PC3). (**d**) Nuclear and cytoplasmic distribution of miR-4534 and isomiR-4534 in PC3 cells. (**e**) miR-4534 and isomiR-4534 expression in DU145 cells 24 h after transfection with pre-miR-4534 (pMIR4534) or control expression vector. (**f**) Expression of miR-4534, isomiR-4534, miR-21 and miR-22 in PC3 cells after transfection of pri-miR-4534 targeting (siprimiR) or control (siGL3) siRNA. (**g**) miR-4534 and isomiR-4534 expression in DU145 cells transfected with pre-miR-4534 targeting gRNA constructs (del91-55 and del88-52) or empty vector and selected with puromycin. (**h**) CDH1 expression in PC3 (left) and LNCaP (right) cells after transfection with miR4534 and isomiR-4534 mimics or negative control (ctrl neg) miRNA. (**i**) CDH1 mRNA (top) and protein (bottom) expression in DU145 cells transfected with pri-miR-4534 targeting siRNA (siprimiR) or siGL3. Data are mean±s.d. of three independent determinations. Student's *t*-test was used for *P* value assessment. *P<0.05.

The formation of the AGO1/S-paRNA complex is a critical step determining the transition from transcriptional activation to repression in CDH1^L^ cells. AGO proteins are guided to their RNA targets by miRNAs^35^,^36^. To identify small RNAs bound to the S-paRNA-AGO1 complex, we combined RIP with biotin-labelled RNA pull-down (Fig. 4a). Control experiments with IgG and monitoring the recovery of biotin-labelled S-paRNA confirmed the efficiency and stringency of the procedure. Cloning and sequencing of the small RNA fraction retrieved a single prominent small RNA in ∼70% of the sequenced clones, indicating a highly specific interaction with the S-paRNA-AGO1 complex (Fig. 4b).

The retrieved small RNA has close similarity with human miR-4534 in miRBase. The two sequences are identical apart from two nucleotide changes (A to C and G to C) and a single-base deletion at the 5′-end. Public small RNA-Seq databases contain numerous instances of small RNAs, frequently more abundant than the canonical miR-4534, with identical or similar sequences to the cloned small RNA and classified as isomiRs of miR-4534 (Supplementary Fig. 5a), suggesting that the AGO1-bound small RNA could be an isomiR of this miRNA. Using selective primers, we detected both the mature miR-4534 and the isomiR-4534 in normal and prostate cancer cell lines (Fig. 4c). IsomiR-4534 is more abundant than miR-4534 in all cell lines tested and its expression is lower in normal PrECs compared to transformed and tumorigenic cell lines. Furthermore, unlike miR-4534, isomiR-4534 is more abundant in nuclei than in cytoplasm of PC3 cells (Fig. 4d).

IsomiRs can be produced by mutations or RNA editing^38^. Sequencing of the pre-miR-4534 locus did not reveal any mutation (Supplementary Fig. 5b), thereby favouring the hypothesis of RNA editing. Interestingly, both canonical (A-to-I) and non-canonical editing of mRNAs and miRNAs has been shown in human cells^39^,^40^,^41^,^42^. Therefore, we examined whether isomiR-4534 could be generated from pre-miRNA-4534. After transfection of pre-miR-4534, we detect a significant increase of miR-4534 and isomiR-4534, consistent with the substantial conversion of pre-miR-4534 to both mature miRNAs (Fig. 4e). Next, we tested the effects of pri-miR-4534 knockdown on isomiR-4534 levels. To this end, we identified the primary transcript of pri-miR-4534 by scanning the region immediately upstream of the pre-miR-4534 locus on chromosome 22 and designed a pri-miR-4534-targeting siRNA, which effectively reduced its level (Supplementary Fig. 5c–e). Knockdown of pri-miR-4534 reduces both miR-4534 and isomiR-4534 indicating that both mature miRNAs originate from the same primary transcript, while unrelated miRNAs, miR-21 and miR-22, were not significantly affected (Fig. 4f). In addition, we used the CRISPR-Cas9-FokI nuclease system^43^,^44^ to genetically modify the pre-miR-4534 locus and prevent production of the mature miRNA. Transfection with the pre-miR-4534 targeting vectors (del91-55 and del88-52) significantly reduces the level of isomiR-4534 compared to control vector transfected cells (Fig. 4g). Single-cell clones derived from cells expressing the pre-miR-4534 targeting constructs show deletions in the pri-miR-4534 locus compatible with single allele inactivation and substantial reduction of isomiR-4534 (Supplementary Fig. 5f,g). Thus, targeting pri/pre-miR-4534 using both siRNA and CRISPR-Cas9 leads to a significant reduction of isomiR-4534. Importantly, transfection of isomiR-4534 reduces CDH1 expression in PC3 cells whereas miR-4534 has no effect (Fig. 4h). Both isomiR-4534 and miR-4534 were ineffective in LNCaP cells. Furthermore, knockdown of pri-miR-4534, which reduces isomiR-4534 level, increased CDH1 expression in PC3 cells (Fig. 4i).

**Figure 5:**
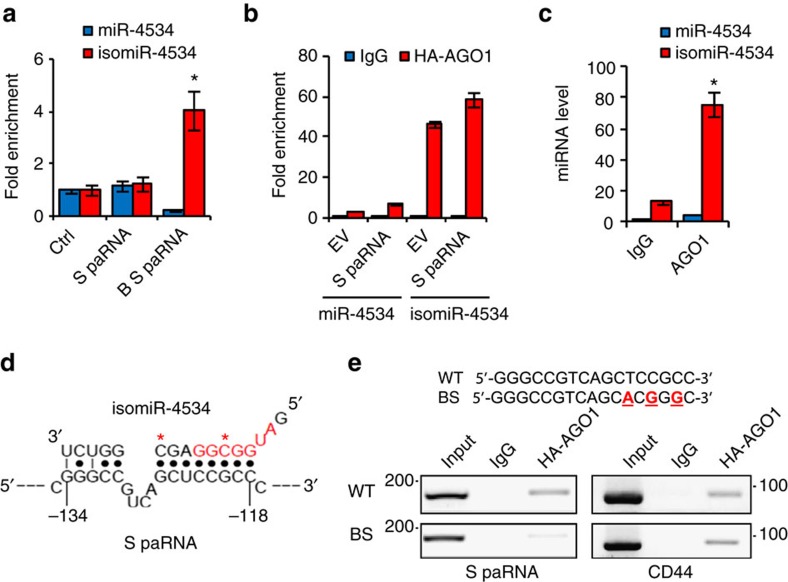
IsomiR-4534 guides Argonaute 1 binding to sense promoter-associated RNA. (**a**) Binding of isomiR-4534 to HA-AGO1 and biotinylated S-paRNA (B S-paRNA) in RNA pull-down. S-paRNA, nonbiotinylated S-paRNA; Ctrl, control RNA. (**b**) IsomiR-4534 binding to HA-AGO1 in cells transfected with empty (EV) or S-paRNA expression vector determined by RIP. (**c**) IsomiR-4534 binding to chromatin-associated AGO1 determined by RNA-ChIP. (**d**) Predicted base pairing of isomiR-4534 with S-paRNA. Asterisks, edited bases in isomiR-4534; Red bases, nucleotides in putative canonical seed region. (**e**) Binding of AGO1 to S-paRNA with either wild-type (WT) or mutated isomiR-4534 binding sequence (BS) determined by RIP. CD44 mRNA, positive control. Top, red underlined bases indicate mutated bases in the BS construct. Data are mean±s.d. of three independent determinations. Student's *t*-test was used for *P* value assessment. *P<0.05.

Collectively, these results support the hypothesis that isomiR-4534, generated by non-canonical editing of pri/pre-miR-4534, guides the formation of a repressive complex on the CDH1 promoter. qRT–PCR after RIP and biotin-labelled RNA pull-down showed the selective association of isomiR-4534 with the AGO1/S paRNA complex (Fig. 5a). In cells expressing HA-AGO1, we detect avid binding of isomiR-4534 to AGO1 by RIP (Fig. 5b). Binding of isomiR-4534 is slightly increased by co-expression of the S-paRNA, while minimal amounts of miR-4534 are associated with AGO1. Moreover, RNA-ChIP demonstrates that isomiR-4534 is associated with endogenous chromatin-bound AGO1 in PC3 cells (Fig. 5c), a result consistent with the formation of a complex between the miRNA-loaded AGO1 and S-paRNA on the CDH1 promoter.

To determine how isomiR-4534 guides AGO1 to the S-paRNA, we interrogated the sequence of the noncoding transcript using miRNA target prediction tools. We identify a single potential target site located at −118 to −134 in the S-paRNA compatible with miRNA binding^45^. A shift in the miRNA seed region allows extended base pairing (8 bp) and the formation of a stable complex with S-paRNA (Δ*G*=−27.2 kcal mol^−1^; Fig. 5d). Conversely, this site is poorly compatible with the sequence of mature miR-4534, which exhibit incomplete base pairing in the seed region and a predicted lower affinity (Δ*G*=−22.8 kcal mol^−1^) (Supplementary Fig. 6a), suggesting that editing modifies substantially the seed region and may direct isomiR-4534 to a distinct repertoire of targets compared to miR-4534. Consistent with this notion, we find a greatly reduced number of predicted targets of isomiR-4534 in canonical 3′-UTR compared to miR-4534 (Supplementary Fig. 6b).

The predicted isomiR-4534 binding sequence is within the AGO1 interacting region of the S-paRNA. Mutations in the isomiR-4534 binding site (BS) reduces AGO1 binding to S-paRNA, demonstrating that the site anchors AGO1 to the transcript (Fig. 5e). Furthermore, BS mutations increase reporter activity compared to the WT reporter (Supplementary Fig. 6c). Interestingly, the BS mutations did not affect reporter activity when introduced in the TS reporter unable to synthesize full length S-paRNA, indicating that the mutations affect primarily the S-transcript ability to bind AGO1 (Supplementary Fig. 6d).

### SNP rs16260 influences AGO1 recruitment to CDH1 promoter

**Figure 6:**
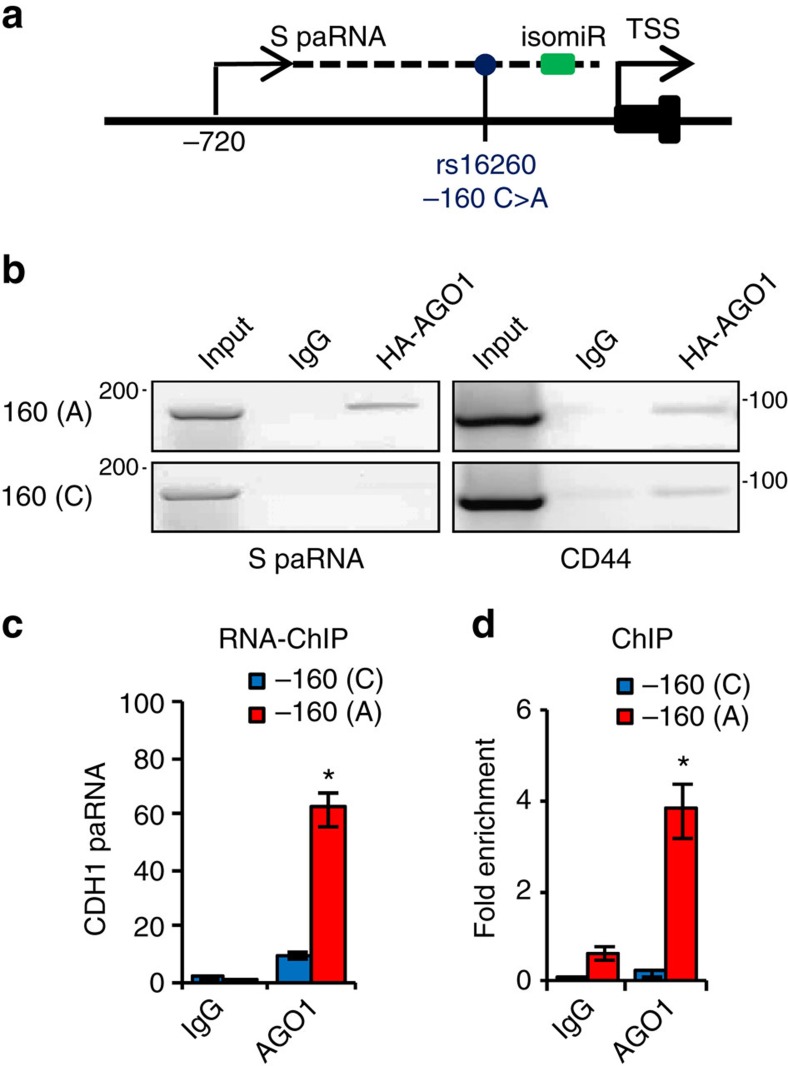
SNP rs16260 affects binding of Argonaute 1 to sense promoter-associated RNA. (**a**) Position of SNP rs16260 (blue circle) and isomiR-4534 binding site (green rectangle) in the CDH1 promoter. (**b**) Allele-specific binding of HA-AGO1 to the −160A and −160C S-paRNA determined by RIP. CD44 mRNA, positive control. (**c**) Allele-specific binding of endogenous AGO1 to the 160A and −160C S-paRNA determined by RNA-ChIP. (**d**) Allele-specific binding of AGO1 to the CDH1 promoter with either the 160A and −160C allele determined by ChIP. Data are mean±s.d. of three independent determinations. Student's *t*-test was used for *P* value assessment. *P<0.05.

SNPs linked to cancer predisposition are present at the CDH1 promoter^46^. We focused on SNP rs16260 (−160C>A) located near the isomir-4534 binding sequence (Fig. 6a). The minor A allele has been associated with increased risk of epithelial cancers, including prostate cancer^46^,^47^,^48^. Furthermore, we found that the A/A genotype is associated with reduced recurrence-free survival after prostatectomy in prostate cancer patients (*P*<0.05; Supplementary Fig. 7a,b), consistent with an impact on CDH1 expression during tumour progression. This SNP has been proposed to affect the binding of unidentified transcription factors to the CDH1 promoter, but no direct evidence was provided^49^.

To investigate whether SNP rs16260 influenced CDH1 promoter activity, we generated reporter constructs with the two allelic variants. The −160A reporter exhibits lower transcriptional activity than the −160C reporter in PC3 cells (Supplementary Fig. 7c). No difference is observed in LNCaP cells, consistent with a cell context-dependent effect of the SNP requiring expression of the S-paRNA. Notably, similar amounts of sense transcripts are produced from the A and C reporters (Supplementary Fig. 7d). Intriguingly, we did not find a clear correlation between the rs16260 genotype and CDH1 expression in cell lines, confirming that the SNP's effect are manifest only in a specific context (Supplementary Fig. 7e).

Based on the proximity to the isomiR-4534 binding sequence, we hypothesized that SNP rs16260 could affect the interaction of AGO1 with the CDH1 promoter. Therefore, we performed RIP with the two S-paRNA allelic variants. AGO1 binds preferentially to the −160A allele (Fig. 6b), also leading to preferential binding of SUV39H1 to (−160A)S-paRNA in the presence of AGO1 (Supplementary Fig. 7f). Importantly, the effect of the SNP depends on the presence of the S-paRNA and are completely independent of promoter DNA.

Using allele-specific RNA-ChIP qPCR in the heterozygous DU145 cells we find that SNP rs16260 has a similar impact on the association of chromatin-bound AGO1 to the CDH1 promoter (Fig. 6c). AGO1 binds almost exclusively to (−160A)S-paRNA with minimal binding to the −160C allele. Consistent with this result, allele-specific ChIP qPCR showed preferential binding of AGO1 to the promoter carrying the −160A allele (Fig. 6d). Thus, SNP rs16260 affects the ability of S-paRNA to bind and recruit AGO1 to the CDH1 promoter. Based on sequencing data, similar amounts of −160A and −160C transcripts are produced in heterozygous DU145 cells (Supplementary Fig. 7g), thus excluding unbalanced synthesis from the two alleles as the cause of the preferential binding of AGO1 and arguing in favour of a qualitative difference between the two allelic variants.

Since SNP rs16260 does not alter the isomiR-4534 binding sequence, we hypothesized that the polymorphic site could influence the assembly of the S-paRNA-AGO1 complex indirectly by altering the transcript secondary structure. Using Mfold, a RNA structure prediction tool, we determined that the minimal AGO1 binding region (−281/−57) of S-transcript folds in a well-defined secondary structure, which is also maintained in the full length (−668/−57) S-paRNA (Supplementary Fig. 8a,b). Interestingly, SNP rs16260 introduces conformational changes, which are limited to the AGO1 binding region and affect the organization of three main stem-loops, one of them encompassing the isomiR-4534 binding site. Analysis of base-pair probability performed with Sfold indicates differences at the isomiR-4534 binding site between the two allelic variants (Supplementary Fig. 8c). We further compared between the two allelic variants the free energy requirement for forcing the hairpin (−138 to −117 nt) including the isomiR-4534 binding site into single-strand conformation and to make it accessible (Supplementary Fig. 9a,b). Unwinding of the hairpin in the (−160A)S-paRNA was significantly facilitated compared to −160C allele (ΔΔ*G*=−9.31 versus −13.33 kcal mol^−1^) (Supplementary Fig. 9c).

**Figure 7:**
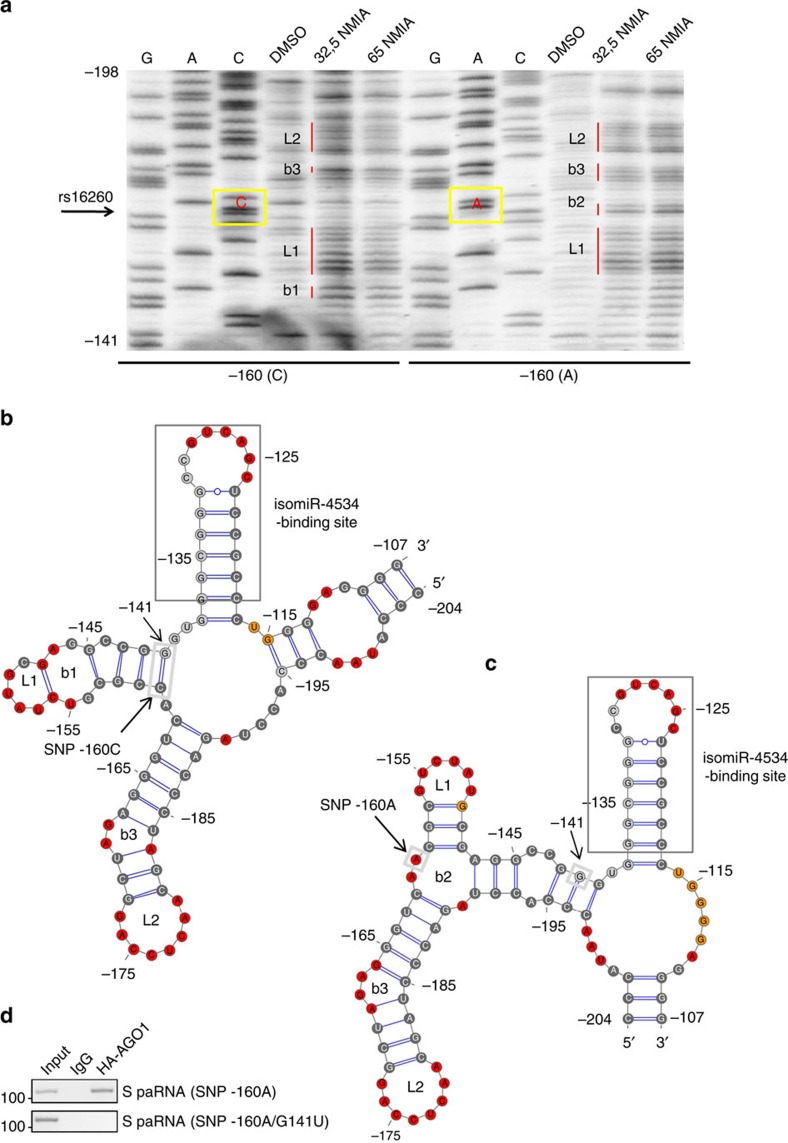
SNP rs16260 influences folding of sense promoter-associated RNA. (**a**) SHAPE reactivity gel analysis of the sense transcript (−688/−57) showing the region encompassing SNP rs16260 with C (left) and A (right) variants. G, A, and C, sequencing lanes. Samples were incubated with 0 (DMSO), 32 or 65 mM NMIA. Yellow boxes, position of the polymorphic site with either C or A variant. (**b**,**c**) Secondary structures of S-paRNA with the −160C (**b**) and −160A (**c**) allele built using RNAstructure based on SHAPE-derived constraints and visualized using VARNA. Colours mark nucleotides that are either unstructured (red, SHAPE ≥0.85), well-ordered (black, SHAPE <0.4), with intermediate reactivity (orange) or non-resolved/undetectable (gray). (**d**) Binding of HA-AGO1 to −160A and mutated −160A/G141U S-paRNA determined by RIP. B, bulges; dark gray box, isomiR-4534 binding site; L, loops; light gray boxes, base pair in −160C allele disrupted in −160A variant.

The secondary structure of the full length S paRNA (−688/−57) with either A or C variants was then experimentally determined using selective 2′-hydroxyl acylation and primer extension (SHAPE)^50^. Consistent with the *in silico* predictions, SHAPE reveals that the S-paRNA is highly structured, including the region near SNP rs16260 (Fig. 7a). The isomiR-4534 binding sequence is partially double-stranded as well, suggesting that access of the AGO1/isomiR-4534 complex is affected by the RNA secondary structure. Furthermore, the A and C variants differ significantly in the pattern of SHAPE reactivity at and around SNP rs16260, revealing a change in the secondary and tertiary structure of the transcript for the two polymorphic sequences (Fig. 7a). Using RNAstructure we generated secondary structure models that were consistent with the SHAPE data and differed significantly between the two variants (Fig. 7b,c). The −160A allele paRNA has increased spacing between loop L1 and the isomiR-4534 -binding site hairpin, which at visual inspection could allow better access to the AGO1-isomiR-4534 complex compared to the −160C allele.

Using the SHAPE-determined structures to estimate the likelihood of the isomiR-paRNA interaction, we find that the C to A exchange at position −160 decreases the base-pairing probability for the isomiR-4534 binding site hairpin from >99% to 90–95%, indicating a greater predisposition of the hairpin to unwind and allow access to the isomiR-4534 by strand invasion (Supplementary Fig. 10). The target accessibility and binding energy for the isomiR-4534-paRNA interaction were further estimated using RNAup. Consistently, the energy requirements for both unwinding of the target (9.6 versus 12.0 kcal mol^−1^) and isomiR-4534 binding (−7.8 versus −5.4 kcal mol^−1^) indicate greater accessibility and binding capacity of the −160A allele (Supplementary Fig. 11). The greater accessibility of the (−160A)S-paRNA to isomiR-4534 would in turn increase its ability to recruit AGO1.

To test directly this hypothesis, we restored the stem-loop conformation observed for the −160C allele by introducing in the (−160A)S-paRNA a single compensatory mutation (G141U) to recreate the base pairing (C160:G141) at the base of the hairpin disrupted in the A variant (A160/G141) (Fig. 7b,c). The G141U mutant allows the base pairing (A160:U141) and establishes a stem-loop structure as observed for the −160C variant (Supplementary Fig. 12). When the G141U mutation is introduced in the (−160A)S-paRNA, binding of AGO1 is substantially reduced, confirming that the transcript secondary structure influences isomiR-4534 access and AGO1 binding (Fig. 7d). Thus, the structural change induced by the −160A allele favours unwinding of the stem-loop and assembly of the isomiR-4534-AGO1complex on the S-paRNA compared to the −160C allele.

## Discussion

This study investigates how promoter-proximal transcripts sustain transcriptional silencing of E-cadherin in cancer cells. Furthermore, the analysis of a cancer-associated genetic polymorphism in the CDH1 promoter uncovers a mechanism by which noncoding genetic variants influence epigenetic processes and affect functional elements in promoter–proximal transcripts by altering RNA secondary structure.

**Figure 8:**
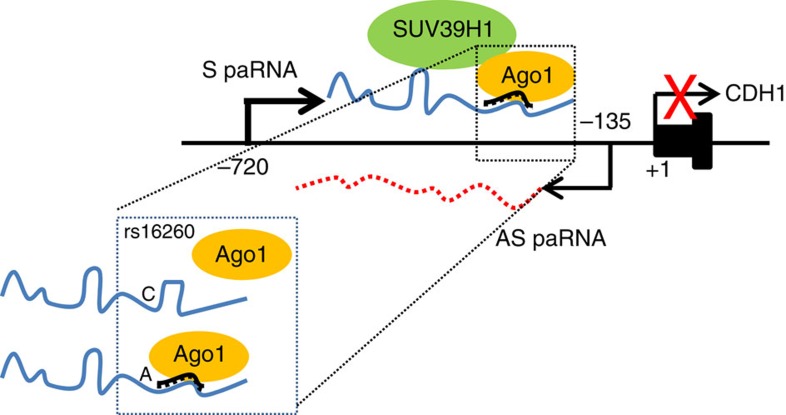
Proposed model of the impact of SNP rs16260 on CDH1 promoter regulation. Assembly of the isomiR-4534/AGO1 complex on sense promoter-associated RNA silences CDH1 transcription. SNP rs16260 influences, through changes in the sense transcript secondary structure, the loading of isomiR-4534/AGO1 and the assembly of the transcriptional silencing complex.

Epigenetic silencing of tumour suppressor genes like CDH1 are key events in tumour development and progression^51^. Here, we show that transcriptional silencing and activation of CDH1 are at least partially controlled by an epigenetic switch that relies on S and AS-transcripts generated from distinct initiation sites upstream of the gene *TSS* (Fig. 8). The transition from the active to the inactive state of the CDH1 promoter is associated both in cell lines and human tumours with changes in the relative abundance of S and AS-paRNAs. Mechanistically, the S-paRNA, which is chromatin-bound and present at very low copy number, cooperates with AGO1 to recruit SUV39H1 to promote a repressive chromatin state. Knock-down of either S-paRNA or AGO1 impairs the binding of SUV39H1 and reactivates CDH1 transcription. Thus, S-paRNA and AGO1 coordinate the access to the promoter of SUV39H1 and possibly other regulatory factors that maintain the repressive state. S and AS-paRNAs are substantially non poly-adenylated, accumulate in nuclei and remain attached to chromatin. Notably, these transcripts originate from distinct and functionally independent initiation sites upstream of the gene TSS which, when inserted in a promoter-less reporter as well as in the context of full length promoter constructs, exhibit striking cell context specificity and directionality, a set of observations consistent with other recent studies of upstream promoter transcripts^25^,^26^,^52^.

Both AGO1 and AGO2 are present in nuclei of mammalian cells and interact with chromatin modifiers, components of the splicing machinery and gene promoters^53^,^54^,^55^. Here, we show that AGO1 binds to the CDH1 promoter and is guided by a miRNA (isomiR-4534) to a specific site on the S-paRNA. This miRNA-guided AGO1 complex represses promoter activity and maintains gene silencing. The novel miRNA (isomiR-4534) identified in this study is generated by non-canonical editing of the pri/pre-miR-4534, is abundant in cancer cells compared to normal epithelial cells and suppresses CDH1 expression when transfected in S-paRNA expressing cells.

The paRNA-based epigenetic network in the CDH1 promoter is influenced by a genetic polymorphism, SNP rs16260, which affects the ability of the S-paRNA to form a complex with miRNA-AGO1 independently of changes in DNA regulatory elements. The −160A allele is associated with increased risk of multiple types of cancer, including prostate cancer^46^,^47^,^48^. We find that the −160A allele increases AGO1 binding in a S-paRNA dependent manner and reduces CDH1 promoter activity. RNA secondary structure prediction analyses and SHAPE show that the AGO1 binding region of the S-paRNA forms three closely-spaced stem-loops that overlap with the isomiR-4534 binding site. The SNP rs16260 causes a change in S-paRNA structure near the −160(C/A) site that extends to the isomiR-4534 target sequence, resulting in changes in the relative distance and local folding of the core hairpins. The structural changes imposed by the SNP −160(C/A) affect the ability of isomiR-4534 and AGO1 to bind to S-paRNA, leading to increased binding of AGO1 to the −160A allele. The increased propensity of the A allelic variant to bind AGO1 would favour silencing of the gene in permissive cells.

In summary, our findings provide new insight on the contribution of paRNAs to the epigenetic machinery and to the impact of genetic variants on gene expression. An increasing number of disease-associated SNPs map to long ncRNAs and are proposed to change their protein binding capability^56^,^57^,^58^,^59^. Our result suggest that altering the sequence, structure and, therefore, function of paRNAs provides an additional and still unexplored way by which polymorphic sites and somatic mutations in noncoding regions influence the epigenetic landscape and impact on human disease. These results also support the development of inhibitors targeting these ncRNAs for gene-selective transcriptional reprogramming^28^,^34^,^60^,^61^, since knockdown of S-paRNA reactivates CDH1 expression in prostate cancer cells and impairs their proliferative, clonogenic and stem-likeness. Reprogramming transcription of individual genes through this approach would have broad implications for epigenetic therapy of cancer and other diseases.

## Methods

### Cell cultures and transfection

Prostate cancer cell lines (PC3, LNCaP and DU145) were obtained from ATCC and maintained in RPMI-1640 (Gibco) supplemented with 10% FBS. Cells lines are characterized by DNA profiling (short tandem repeat analysis). Cells were used within 6 months of culturing and regularly checked for Mycoplasma contamination using the MycoAlert Mycoplasma Detection Kit (Lonza). Normal (PrEC) and transformed (EHFkd-PrEC) prostate epithelial cells were previously described^24^. EHFkd-PrEC cells were generated by stable transfection of PrEC with ESE3/EHF-directed short hairpin RNAs (shRNA). Both PrEC and EHFkd-PrEC were maintained in serum-free complete Prostate Epithelial Growth Medium (PrEGM; Cambrex, Lonza), which is supplemented with Bovine Pituitary Extract (BPE), hydrocortisone, Human Epidermal Growth Factor (hEGF), epinephrine, insulin, triiodothyronine, transferrin, gentamicin/amphotericin-B, and retinoic acid.

siRNAs, including Silencer Negative Control (siControl), were purchased from Ambion. siRNA sequences are shown in Supplementary Table 1. S-paRNA targeting siRNAs were selected using Sfold to ensure maximum target accessibility and strand-specificity towards the sense transcript, based on the average unpaired probability of the targeted sequence and asymmetry of the base composition (differential stability of siRNA duplex ends, DSSE=>1 kcal mol^−1^). Cells were transfected with siRNAs using Lipofectamine2000 (Invitrogen) or Interferin (Polyplus)^60^. Plasmids were transfected with JetPRIME (Polyplus). To assess the effects on cell proliferation, colony formation and tumour sphere formation, cells were transfected with the indicated siRNAs (100 nM) and 24–72 h later detached, counted and reseeded as previously described^34^,^60^. For cell proliferation assays cells were plated in 12-well plates with four replicates per experimental group. At the indicated time points viable cell number was determined by trypan blue counting. For colony assay cells were plated in triplicate in 6-well plates (1,000 cells per well). After 12–14 days, cells were fixed and stained with 1% crystal violet in 20% ethanol. Colonies were counted with an automated colony counter (Alphaimager). To assess tumour sphere formation cells were plated in ultra-low attachment dishes (Corning Life Sciences) in serum-free Mammary Epithelial Basal Medium (MEBM, Cambrex). Tumour spheres (≥50 μm in diameter) were counted after 8–10 days.

### RNA and SNP analysis in tissue samples

Tissue samples were collected with approval of the Ethical Committee of the Piedmont Region, Italy, and written informed consent from patients undergone radical prostatectomy for localized prostate cancer^62^. Total RNA was extracted from snap frozen tumour samples (*n*=24) using SV Total RNA Isolation System (Promega) and analysed by RT–qPCR or strand-specific RT–qPCR. Primers (paRNA, CDH1 −282F and −171R; CDH1 mRNA, CDH1 +2497F and +2619R) are shown in Supplementary Table 2. For genotyping by restriction fragment length polymorphism (RFLP) analysis or sequencing, genomic DNA was extracted from two 10-μm sections of formalin-fixed paraffin embedded (FFPE) prostate tumour specimens (*n*=47) using DNeasy Tissue and Blood kit (Qiagen). Genomic DNA was amplified by PCR along with positive (Male DNA Universal Reference) and negative control (No template) reactions using primers (CDH1 −272F and −82R). PCR products (5 μl for cell lines and 15 μl for FFPE samples) were digested with HincII (TaKaRa) and analysed by agarose gel electrophoresis. SNP analysis in cell lines was performed by RFLP and sequencing. The relationship between SNP rs16260 and recurrence-free survival was examined in a patient cohort with available pathological and clinical follow up data^24^.

### Promoter-associated transcript prediction

Raw data from global nuclear run-on sequencing (GRO-Seq) performed in various cell lines were downloaded. Source of the data were for LNCaP cells (series GSE47805, sample GSM1159895)^20^, MCF7 cells (series GSE45822, samples GSM1115995, GSM1115996, GSM1115997 and GSM1115998)^21^, HCT116 cells (series GSE53964, samples GSM1304424, GSM1366021 and GSM1304426)^22^ and h1ESC cells (series GSE41009, samples GSM1006729 and GSM1006731)^23^. The raw reads in fastq files were extracted and aligned with bowtie2 (version 2.2.1)^63^ allowing up to 1 mismatch and accepting uniquely mapping reads on the reference genome GRCh37.p13 provided by Genecode. Samtools (version 0.1.19)^64^ was used to convert sam to bam files and sort them. Using HOMER (Hypergeometric Optimization of Motif EnRichment) suite of tools for Motif Discovery and next-generation sequencing analysis^19^ we predicted genome-wide the transcription events for each cell lines (‘findPeaks.pl') allowing a minimum size of 50 bp for transcript body detection. Only transcripts identified within 2 kb upstream to TSS of annotated genes were considered as promoter-proximal transcripts. The predicted promoter-associated transcripts were annotated as S and AS relative to the direction of the adjacent gene (Supplementary Table 3). Quantification of rpkm values of annotated and novel transcripts was performed with ‘analyzeRepeats.pl' included in HOMER suite. To visualize GRO-Seq data Integrative Genomics Viewer (IGV) was used (version 2.3.32).

### Cloning and site-directed mutagenesis

pIRESneo-FLAG/HA-AGO1 expression vectors was obtained from Addgene. pGL3-CDH1 promoter reporter was provided by Dr H. Crawford and Myc-tagged SUV39H1 was provided by Dr D. Reinberg. pGL3 basic and pRL-SV40 were obtained from Promega. pcDNA3.1(+/−) from Invitrogen. CDH1 promoter fragments were amplified from pGL3-CDH1 promoter (−790/+48) using AmpliTaq Gold Polymerase (Roche). Promoter fragments −790/−709 (S-TSS) and −57/−280 (AS-TSS) were subcloned in pGL3-basic using KpnI/HindIII and HindIII/SacI restriction sites, respectively. Promoter fragment −373/+48 was subcloned in pGL3-basic using KpnI and BglII restriction sites. Promoter fragments −668/−57, −476/−57, −668/−261 and −281/−57 were cloned into pcDNA3.1(+/−) plasmid either in sense or antisense orientation using either EcoRI and ApaI or BamHI and ApaI restrictions sites. Site-directed mutagenesis was performed with Invitrogen Mutagenesis System Kit using either pGL3 or pcDNA3.1 vectors as templates. Primers for PCR amplification and site-directed mutagenesis are shown in Supplementary Table 2. *Apa*I and *Eco*RI restriction sites were introduced by site-directed mutagenesis at position −668 of the pGL3-CDH1 promoter to produce the pGL3-CDH1-T1 promoter vector. To generate the pGL3-CDH1-TS promoter vector the transcription termination sequence (SV40 polyA site) in the pGL3-basic plasmid was PCR amplified and cloned in the ApaI-EcoRI sites of pGL3-CDH1-T1 promoter vector. Plasmids were amplified in JM109 or DH5α competent cells and purified by GenElute Plasmid Miniprep Kit (Sigma). DNA sequences of all constructs were confirmed by DNA sequencing.

### Luciferase reporter assay

Luciferase promoter reporter vectors (100–200 ng) were transfected along with pRL-SV40 Renilla luciferase reporter (10 ng) in cells plated in 48-well plates. After 48 h Firefly and Renilla luciferase activities were measured using Dual Luciferase assay kit (Promega). Renilla luciferase was used to normalize for transfection efficiency.

### RNA extraction

Total RNA was extracted from cell lines using SV Total RNA Isolation System (Promega). RNA samples were treated with DNase I (Promega) to remove genomic DNA. For miRNA expression analysis RNA was purified by phenol-chloroform extraction. To examine intracellular distribution of promoter-associated transcripts cells were lysed in 100 mM Tris HCl (pH 7.4), 100 mM NaCl, 2.5 mM MgCl_2_, 40 μg ml^−1^ Digitonin (RSB-100)^65^. After centrifugation the supernatant containing cytosolic RNA was collected. Pellets were suspended in RSB-100 with 0.5% Triton X (RSB-100T) and after centrifugation the supernatant containing nuclear RNA was recovered. The final pellets were suspended in RSB-100T, sonicated and centrifuged. The supernatant containing chromatin-bound RNA was recovered. RNA was extracted from all the collected fractions using TriReagent (Invitrogen).

### 5′ and 3′ rapid amplification of cDNA ends

5′ RACE was performed with gene-specific primers for the sense and antisense transcripts (Supplementary Table 2) using Invitrogen 5′ RACE System and RNA from PC3 and LNCaP cells. cDNA was purified, tailed with dCTP and amplified consecutively with gene specific primers and either Abridged Anchor primer or Abridged Universal Amplification primer provided in the 5′RACE system kit. For 3′RACE, nuclear and chromatin-associated RNA was polyadenylated with Poly(A) tailing kit (Applied Biosystem). Polyadenylated RNA was then retro-transcribed and amplified consecutively with gene-specific primers using Invitrogen 3′ RACE system kit. Final PCR products were cloned into pGEM-T Easy vector (Promega) and sequenced.

### Polymerase chain reaction

RT–PCR was performed using SuperScript One Step RT-PCR system (Invitrogen). Samples were analysed by agarose gel electrophoresis followed by staining with GelRed (Biotium) and imaging with AlphaImager (Innotech) with primers (paRNA, CDH1 −303F and −153R; CDH1 mRNA, CDH1 +610F and +810R) (Supplementary Table 2). Densitometry was performed using the AlphaImager software. Uncropped images of the gels are shown in Supplementary Figs 13–17. For strand-specific RT-PCR either the forward or reverse primers were added separately to the reverse transcriptase reaction. Both for RT-PCR and strand-specific RT-PCR, reactions in which the reverse transcriptase step, PCR primers or template RNA were omitted were run in parallel to exclude presence of genomic DNA and self-priming. To confirm specific RNA amplification, DNase-treated RNA extracted from cells transfected with expression vectors was incubated with or without RNase A (1 μg, USB) for 10 min at 37 °C and before RT-PCR. Quantitative RT-PCR (qRT-PCR) was performed using EXPRESS-One step SYBR GreenER (Invitrogen) on ABI Step One Plus (Applied Biosystems) and primers for paRNA (CDH1 −303F and −153R) and CDH1 mRNA (CDH1 +2,497F and +2,619R). Strand-specific qRT-PCR was performed using Power SYBR Green One-step RNA-to-Ct system (Invitrogen) using either forward or reverse primers in the reverse transcriptase step. qRT–PCR data were analysed using standard curves and normalized to β-actin as reference gene. Quantitative real-time PCR (qPCR) using genomic DNA as template was performed using SYBR Green FAST qPCR (KAPA Biosystem).

### Allele-specific qPCR

Allele-specific qPCR was performed using SYBR FAST qPCR (KAPA biosystem) and allele-specific primers (CDH1 −180_AF, −180_CF and −57R) (Supplementary Table 2). Forward primers contained an internal mismatch adjacent to the nucleotide complementary to the polymorphic site as this was shown to increase allele discrimination^66^. To increase sensitivity a first round of PCR or RT-PCR using DNA or RNA template was performed with primers flanking the site of interest (CDH1 −476F and −57R). Samples were then diluted and amplified with internal allele-specific primers. Conditions for allele-specific qPCR were determined by testing serial dilutions of first round PCR products and genomic DNA from homozygous and heterozygous cells to ensure efficiency and selectivity of the method. Data were analysed using the standard curve method.

### MicroRNA quantification

Total RNA was retro-transcribed using TaqMan MicroRNA Reverse Transcription Kit (Applied Biosystem). The resulting cDNA was used as template to measure miR-4534, miR21 and U6 snRNA using pre-designed TaqMan Small RNA Assays and a custom made TaqMan assay for isomiR-4534 (Applied Biosystem). Data were analysed using either the ΔΔCt or standard curve method as indicated.

### DNA and RNA sequencing

DNA or RNA was amplified by PCR or directional RT–PCR, respectively, with the indicated primers for the CDH1 and paRNA (CDH1 −281F, −139R) and pre-miR-4534 (pre-miR −194F, +99R) (Supplementary Table 2). After gel electrophoresis, PCR products were purified using Qiaquick PCR purification system (Qiagen) and sequenced.

### Immunoblotting

Cells were lysed in 2.5% SDS and 250 mM Tris-HCl, pH 7.4, at 95 °C. For cell fractionation experiments cells were collected using NE-PER Kit (Pierce) and fractions collected according to manufacturer's instructions. Gel electrophoresis and immunoblotting were done as described^24^. Protein samples were quantified using BCA Protein Assay Kit (Pierce) and 40 μg of protein we loaded in 10%-12% polyacrylamide-SDS gels for electrophoresis. Proteins were then transferred to nitrocellulose membranes for immunoblotting. Membranes were blocked for 1 h with I-block (Applied Biosystem) and then probed overnight at 4 °C with the indicated antibody. Immunoblots were developed using antibodies directed to E-cadherin (BD Biosciences, 610182,1:5,000), β-tubulin (Santa Cruz, sc5236, 1:2,000), GAPDH (Millipore, MAB374, 1:2,000) and anti-mouse (Amersham, NA931-1ML, 1:10,000) or anti-rabbit (Amersham, NA934-1ML, 1:20,000) secondary antibodies. The antibody directed to AGO1 (1:400) was provided by Dr M. Siomi^67^. Uncropped images of the blots are shown in Supplementary Figs 13–17.

### Chromatin immunoprecipitation

Chromatin was prepared and processed as described^60^. Briefly, cells in complete RPMI with 10% FBS were exposed to 1% formaldehyde for 10 min. Cells were then washed and lysed in SDS lysis buffer (5 × 10^6^ cells per ml). Cell lysates were sonicated to shear chromatin and obtain DNA fragments in the range of 200–500 bp. After sonication, the supernatants containing the shared chromatin were recovered by centrifugation and divided in 50-μl aliquots each with 5 × 10^5^ cell equivalents. Each 50-μl aliquot was diluted in a final volume of 500 μl of dilution buffer containing 20 μl of protein A magnetic beads (Upstate). Before the addition of the beads, 10% of each diluted chromatin sample was preserved as Input. Each sample aliquot was incubated overnight at 4 °C with the appropriate antibody. Antibodies for histone H3 dimethylated lysine 9 (H3K9me2, 07–441 EMD Millipore, 8 μg), histone H3 trimethylated lysine 27 (H3K27me3, 07–449 EMD Millipore 5 μg), acetylated histone H3 (H3Ac, 06–599 EMD Millipore, 5 μg), SUV39H1 (Millipore, 05–615, 5 μg) and AGO1 (Merck Millipore, 03–249, 5 μg), and anti-HA (Santa Cruz, F7 SC-7392, 5 μg) were used for immunoprecipitation.

Immunoprecipitates were washed four times using a magnetic separator. After the last wash, both immunoprecipitates and input samples were suspended in 100 μl of elution buffer containing 200 mM NaCl and incubated at 65 °C for 4 h to reverse crosslinking. Next, 4 μl of 1 M Tris-Cl, pH 6.5, 2 μl of 0.5 M EDTA and 1 μg of Proteinase K were added to the samples which were then incubated at 42 °C for 45 min. Magnetic beads were removed and DNA was purified using QIAquick PCR purification KIT (Qiagen). qPCR (primers CDH1 −281F and −139R) was performed as described above. The amount of input and immunoprecipitated DNA was calculated in reference to standard curves. Data are presented as fraction of immunoprecipitated DNA relative to the amount of input DNA in each sample.

### RNA immunoprecipitation

Cells transiently transfected with HA-AGO1, Myc-SUV39H1 and/or paRNA expression vectors were collected after 48 h and processed using Magna RIP RNA Binding Protein Immunoprecipitation Kit (Millipore). Samples were incubated overnight with Magnetic Beads Protein A/G coupled with antibodies directed to HA-tag (F7, Santa Cruz, F7 SC-7392, 5 μg) or Myc (Santa Cruz, A14 SC-789, 5 μg). Beads were washed and RNA was extracted using SV Total RNA Isolation System (Promega). An additional step of DNase I digestion (Qiagen) was performed to remove any plasmid or genomic DNA. Samples were analysed by end-point or quantitative RT–PCR as described above. CD44 mRNA, an AGO1-bound transcript^54^, was used as positive control in RIP experiments with transiently transfected cells. To examine the association of endogenous AGO1 to the CDH1 promoter by RIP, nuclei were isolated from non-transfected cells using NE-PER Kit (Pierce) and then processed using Magna RIP kit and Magnetic Beads Protein A/G (Millipore) coupled with anti-AGO1 (RIPAb+ Ago1 Antibody, 03–249—EMD Millipore, 5 μg) antibody.

### RNA chromatin immunoprecipitation

For RNA-ChIP we followed the ChIP protocol described above except that RNAseOUT (40 U, Invitrogen) was added to the lysis and binding buffers and DNase I digestion was performed before adding antibodies. Reversal of cross-linking was reduced to 2 h (ref. 68). Beads were washed and RNA was extracted and analysed by end point RT–PCR or qRT–PCR as described above.

### Identification of small RNAs

Sense promoter-associated RNA was synthesized by *in vitro* transcription using T7 RNA Polymerase (Roche) and the pGEM-CDH1 promoter vector (−668/−57 fragment) as template. Production of the correct transcript was verified by denaturating polyacrylamide gel electrophoresis. *In vitro* transcribed RNA (50 nmol) was biotinylated using RNA 3′ End Biotinylation Kit (Pierce) according to manufacturer's instructions. Biotin-labelled S-paRNA was purified by phenol-chloroform extraction followed by DTR desalting columns (Edge Biosystems) to eliminate free biotin moieties. PC3 cells were transfected with pIRESneo-FLAG/HA-AGO1 plasmid (2 μg) and 10 pmol of *in vitro* transcribed biotin-labelled S-paRNA using Lipofectamine. Control samples included cells transfected with *in vitro* transcribed non-biotinylated S-paRNA and an unrelated control RNA (pcDNA3.1 plasmid) as well as RIP with control IgG. Cells were collected after 24 h and subjected to RIP as described with minor modifications^69^. Briefly, cells were crosslinked with 1% formaldehyde, washed and lysed in RIP lysis buffer (Hepes 50 mM, EDTA 1 mM, Triton-X 1%) containing RNAseOUT (40 U, Invitrogen). Lysates were then sonicated and treated with DNAse in presence of 25 mM MgCl_2_ and 5 mM CaCl_2._ Cell lysates were incubated for 2 h at 4 °C with 5 μg of anti-HA antibody (F7, Santa Cruz, F7 SC-7392) or control IgG and 20 μl of magnetic beads (Millipore). Magnetic beads (Millipore) were washed and RNA was eluted in 100 mM Tris HCl, pH 7.8, 10 mM EDTA, 1% SDS. Samples were diluted in 10 mM Tris HCl, pH 7.5, 1 mM EDTA, 100 mM NaCl (TEN-100) and incubated with Streptavidin magnetic beads (Roche) for 30 minutes at room temperature. After washing with 10 mM Tris HCl, pH 7.5, 1 mM EDTA, 1 M NaCl (TEN-1000), RNA was eluted by heating at 65 °C for 10 min in 10 mM Tris HCl, pH 6.0, 1 mM EDTA, 2 M NaCl. Samples were digested with proteinase K for 1 h at 42 °C and 1 h at 65 °C. RNA was then extracted using TriReagent. Small RNAs associated with the AGO1 and S-paRNA complex were purified and cloned using miRCAT Small RNA Cloning Kit (Integrated DNA Technologies) according to the manufacturer's protocol. A total of 39 clones were sequenced and 9 of 13 clones with inserted sequences (70%) had the identical sequence that we identified as isomiR-4534. Experiments with control IgG and monitoring of recovery of biotin-labelled S-paRNA confirmed the specificity and stringency of the procedure. The amount of biotin-labelled S-paRNA recovered after RIP with control IgG was minimal. Therefore, cloning and sequencing of the small RNAs fraction in this condition was unlikely to provide information on the S-paRNA-bound small RNA fraction. Similarly, RNA pull-down of samples containing non-biotin labelled RNA or control RNA did not yield detectable amounts of biotin-labelled S-paRNA. To assess recovery of biotinylated S-paRNA by streptavidin-affinity purification an aliquot of the samples was resuspended in 1 M NaCl, 5 mM EDTA, 50 mM MOPS, pH 7.4, 2 M β-mercaptoethanol, heated for 3 minutes at 95 °C to detach from biotin-labelled RNA from streptavidin beads. RNA was extracted with TriReagent and then RT-PCR was performed to detect S-paRNA.

### CRISPR-Cas9-FokI induced mutagenesis

Multiplex gRNA target site oligonucleotides for miR-4534 locus were designed by Zifit software (http://zifit.partners.org). Two different couples of targeting oligoduplexes (pre-miR-4534-91 and pre-miR-4534-55; pre-miR-4534-88 and pre-miR-4534-52) and a middle ultramer duplex were synthesized (IDT, Integrated DNA Technologies). Oligomers were cloned in pSQT1313 (Addgene) to produce the targeting constructs pSQT1313 del-91-55 and pSQT1313 del-88-52. pCAG-hCys4-T2A-nls-hFokI-dCas9-nls (pSQT1601, Addgene) along with pSQT1313 del91-55, pSQT1313 del88-52 or empty pSQT1313 (ratio 3:1) were co-transfected in DU145 cells using JetPrime (Polyplus). pEZX-MR04 (Clonetech) was added (1% of total DNA) to the transfection mix for puromycin resistance. Three days after transfection cells were put under selection (Puromycin, Sigma, 0.5 μg ml^−1^). RNA was extracted after 2–4 days to assess miRNA expression. Aliquots of transfected cells were reseeded in 96-well plates to isolate single cell clones by limiting dilution. Single cell clones that were able to expand were collected and analysed for genomic indels and miRNA levels. DNA was obtained from a total of 37 clones derived from cells transfected with FokI-dCas9 and pSQT1313 d91–55 (*n*=11), pSQT1313 d88-52 (*n*=18) or pSQT1313empty (*n*=8). DNA was amplified with primers for the miR-4534 locus (MIR4534-328 Fw, MIR4534+99 Rev; Supplementary Table 2). PCR products were purified using Qiaquick PCR purification system (Qiagen) and sequenced. Chromatograms were analysed by Mutation Surveyor (Softgenetics) to detect indels and perform trace deconvolution.

### MicroRNA and isomiR database search

miRBase (www.mirbase.org) was used to search for mature miRNAs or premiRNAs (stem loop sequences) with identity or similarity to the cloned AGO1/S-paRNA associated small RNA using BLASTN and SSEARCH methods, respectively. Small RNA-seq databases were searched for miR-4534 and related isomiR sequences. miRGator^70^ and YM500 (ref. 71) include deep sequencing data from human samples collected from GEO, SRA and TCGA archives. Using the provided software we retrieved all isomiRs with up to two mismatches relative to the genomic sequence of mature miR-4534.

### RNA secondary structure and microRNA target prediction

Mfold^72^ was used to predict secondary structure of S-paRNA, base-pair probability and the influence of rs16260 on local folding structure. Forced and non-forced hairpin structures (consecutive bases from −138 to −117) were generated using Mfold by introducing the matrix (P 147 0 16). Accessibility of S-paRNA target region to isomiR-4534 was examined using RNAup (RNAfold website). The analysis of the hairpin probability profile (H-plot) of the S-paRNA allelic variants was done using Sfold. Diana MicroT^73^ and TargetScan^74^ were used to identify putative targets of isomiR-4534 and miR-4534 in 3′UTR of mRNAs. RNAhybrid^75^ and STarMir, an application module of the Sfold software^76^, were used to identify potential binding sequences of isomiR-4534 and miR-4534 in the S-paRNA.

### *In vitro* transcription

pcDNA+ plasmids with −668/−57 (A or C), −476/−57 promoter fragments were linearized with the 5′ overhang 3′ termination restriction enzyme PspOMI (New England Biolabs) or, in case of the −281/−57, construct amplified from the plasmid with the set of primers (SHAPE, pcDNA −120—paRNA F and pcDNA−paRNA +61 polyA R) (Supplementary Table 2). Digested plasmids or PCR amplified fragments were confirmed on a 1% agarose gel and purified with phenol-chloroform extraction followed by ethanol glycogen precipitation or with E.Z.N.A. Cycle Pure Kit (Omega), respectively. Purified DNA templates were used to perform T7 *in vitro* transcription (600 ng per reaction for PCR fragment and 200–600 ng for linearized plasmid) using increasing Mg^2+^ concentrations to optimize conditions. Transcription solutions were incubated at 37 ° C for 3–4 h and the reaction was aborted with 5 μl of 0.5 M EDTA. Transcribed RNAs size and homogeneity were confirmed on 3.5–5% denaturing urea polyacrylamide gel. Positive RNA reactions were pooled together and RNAs were purified by phenol:chloroform extraction followed by precipitation (for each 30 μl reaction) with 70 μl H_2_O, 5 μl of NaCl, 1 μl of 20 μg μl^−1^ glycogen, 2 μl of 100 mM EDTA, 350 μl of cold 100% ethanol, stored at −80 ° C for 30 min, and then centrifuged for 20 min at room temperature at 20,000*g*. The supernatant was removed and the pellet was allowed to air dry for 1 h. Pellets were dissolved to a final concentration of 1 μM in H_2_O DEPC.

### Selective 2′-hydroxyl acylation analysed by primer extension

RNA was labelled with N-methylisatoic anhydride (NMIA) for selective 2′-hydroxyl acylation analysed by primer extension (SHAPE). RNA (36 μl of 1 μM stock) was combined with 20 μl of H_2_O, followed by addition of 56 μl of 2 × folding buffer (200 mM NaCl, 100 mM HEPES, 0.2 mM EDTA, pH 8.0). The reaction was heated at 95 °C for 3 min, and then rapidly cooled on ice for 5 min. The RNA was refolded with the addition of 50 μl of refolding buffer (100 mM NaCl, 50 mM HEPES, 16.5 mM MgCl_2_, pH 8.0). RNA solution was then distributed in 27 μl aliquots into 6 different tubes. 3 μl of DMSO, 32.5 mM NMIA or 65 mM NMIA dissolved in 100% DMSO were added to two of the six tubes. The reactions were heated at 37 °C for 45 min, followed by ETOH precipitation. RNA pellets were resuspended in 15 μl of H_2_O treated with DEPC. Dephosphorylated primers (SHAPE, extREV9, extREV8 extREV5_A extREV5_C) (Supplementary Table 2) were diluted to 3 μM and 5′ labelled with γ^32^ATP (2 μl PNK Buffer A (New England Biolabs, NEB), 3 μl of γ^32^ATP (PerkinElmer, 10 mCi ml^−1^), 9 μl H_2_O, 1 μl PNK enzyme (New England Biolabs). Reactions were incubated at 37 °C for 30 min, followed by the addition of 1 μl of 0.5 mM EDTA, pH 8.0, and incubated at 75 °C for an additional 10 min. Labelled primers were purified with QIAquick Nucleotide Removal Kit (Qiagen) and eluted with 50 μl of water, bringing the final concentration to 0.3 μM. For 3' extension reactions, sequencing reactions (1 μl of unmodified 1 μM RNA combined with 2.5 μll of RNAse free water and 1 pmol of γ^32^ATP labelled primer) and SHAPE reactions (5 μl of modified or DMSO treated RNA (∼6 pmol) combined with 1 pmol of γ^32^ATP labelled primer) were each added with 6.1 and 8.1 μl, respectively, of the enzyme mix. For sequencing reactions additional 4.5 μl ddNTP (5 mM) (either A, G, C, or T) was added to the solution. The reactions were incubated at 52 °C for 1 min, and then 0.5 μl of SuperScript reverse transcriptase III (Invitrogen) was added to each reaction. The reactions were incubated for 10 min at 52 °C, followed by the addition of 0.6 μl of 4 M NaOH. The reactions were heated for 5 min at 95 °C. Following heating, 14.5 μl of acid stop solution (432 μl 2X Urea Loading Buffer, 64 μl 1 M Tris) was added to each reaction, heated at 95 °C for 5 min and then allowed to cool at room temperature. Sequencing and SHAPE reactions (8 μl loaded) were analysed on 8% acrylamide gels (17.5 ml H_2_O, 5 ml 10 X TBE, 10 ml 40% 19:1 bis-acrylamide, 24 g urea, 500 μl 10% ammonium persulfate, 50 μl TEMED). Gels were run for different time at 70 W. Gels were then dried and exposed to a phosphor plate overnight. The phosphor plates were scanned using a Typhoon 600 imager.

### SHAPE quantification and structure modelling

SHAPE data were analysed and quantified using Semi-Automated Footprinting Analysis (SAFA) program^77^. Reactivity data were then incorporated as a shape constraint file in the RNAstructure folding program (http://rna.urmc.rochester.edu/), and the 20 lowest energy structures based on those constraints were generated. The RNA structure models have been visualized using the Visualization Applet for RNA (VARNA) program (http://varna.lri.fr/).

### Statistical analysis

Statistical analyses and custom scripts were performed on R (version R-3.0.3). Kaplan–Meier survival curves were performed using Stata and JMP licensed software.

### Data availability

GRO-seq data sets used in the study can be retrieved from the Gene Expression Omnibus (GEO) with the accession numbers GSE47805 (sample GSM1159895); GSE45822 (samples GSM1115995, GSM1115996, GSM1115997, GSM1115998); GSE53964 (samples GSM1304424, GSM1366021, GSM1304426); GSE41009 (samples GSM1006729, GSM1006731). Genomic coordinates of predicted paRNAs predicted in the CDH1 promoter and genome-wide are available at https://figshare.com: doi.org/10.6084/m9.figshare.4858385.v2 (paRNAs in the CDH1 promoter) and doi.org/10.6084/m9.figshare.4856630.v2 (genome-wide predicted paRNA). All other data that support the findings of this study are available from the corresponding author on request.

## Additional information

**How to cite this article:** Pisignano, G. *et al*. A promoter-proximal transcript targeted by genetic polymorphism controls E-cadherin silencing in human cancers. *Nat. Commun.* **8**, 15622 doi: 10.1038/ncomms15622 (2017).

**Publisher's note:** Springer Nature remains neutral with regard to jurisdictional claims in published maps and institutional affiliations.

## Supplementary Material

Supplementary Information Supplementary Figures and Supplementary Tables
