# Supplementary material for: A promoter-proximal transcript targeted by genetic polymorphism controls E-cadherin silencing in human cancers
Source: Nat Commun. 2017 May 30;8:15622. doi: 10.1038/ncomms15622 (PMC5459991; doi:10.1038/ncomms15622)
Supplement: Supplementary Information — Supplementary Figures and Supplementary Tables [file ncomms15622-s1.pdf]

a

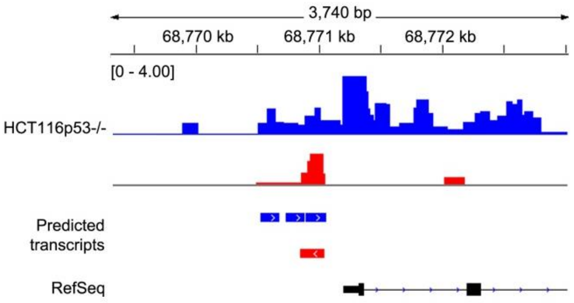

b

Cell line	CDH1 mRNA	AS-paRNA	S-paRNA
HCT116 P53 +/+	390.47	+	+
HCT116 P53 -/-	350.36	+	+
LNCAP	1562.40	+	-
MCF7 C1	1864.76	+	-
MCF7 C2	1812.81	+	-

Supplementary Figure 1. Promoter-associated transcripts at the CDH1 gene locus. (**a**) Transcriptional landscape in the CDH1 promoter in HCT116 p53 -/- cells based on GRO-Seq data. Blue and red tracks, S and AS transcription, respectively. Blue and red boxes, predicted S- and AS-paRNAs. (**b**) CDH1 mRNA expression (normalized read counts) and presence of AS- and S-paRNA from GRO-seq data analysis in the indicated cell lines.

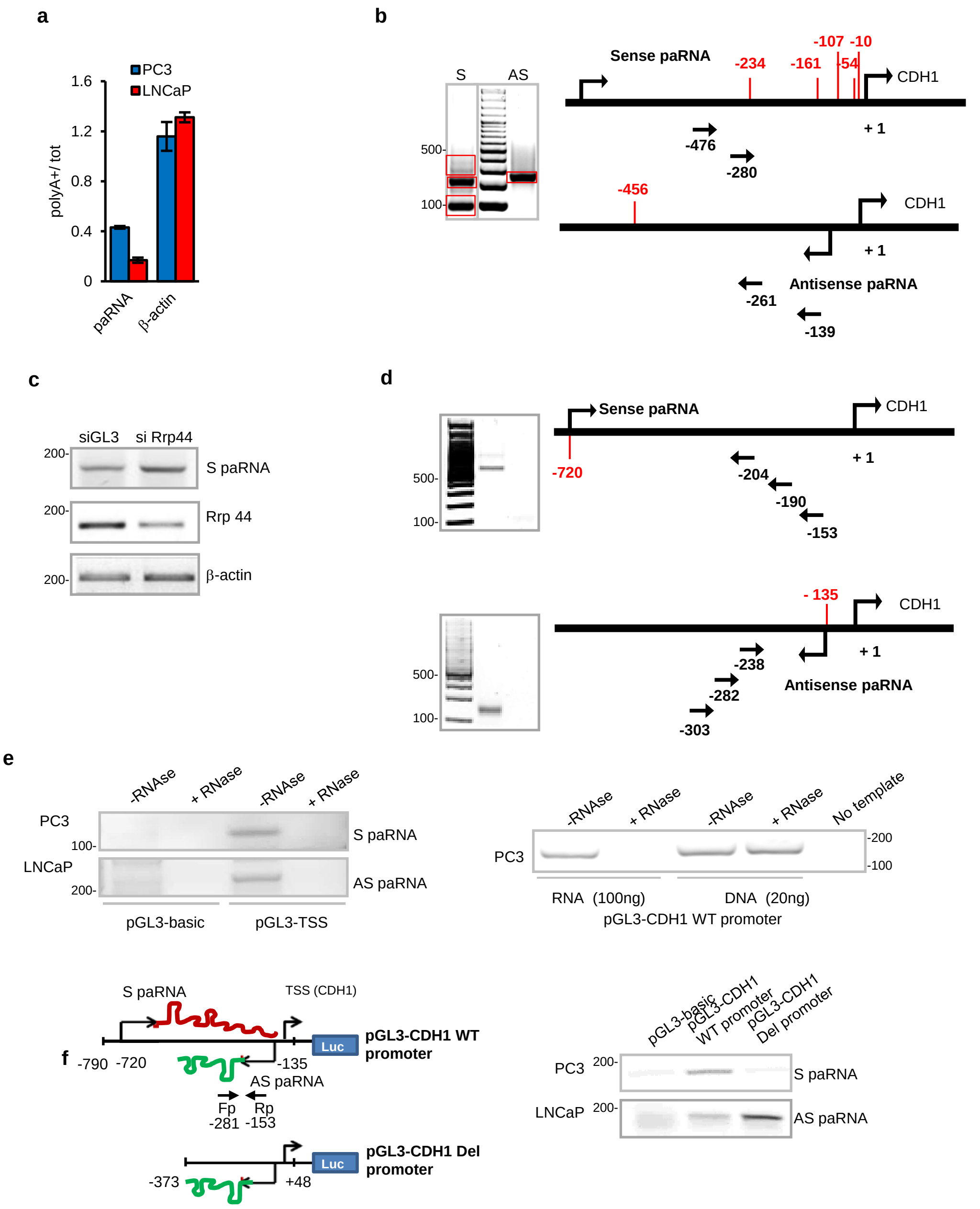

Supplementary Figure 2. (**a**) Poly-adenylation of paRNAs in PC3 (S paRNA) and LNCaP (AS paRNA) cells analysed by differential retro-transcription with random hexamers (total RNA) and oligo dT (polyA+ RNA). β-actin mRNA was used as positive control for a polyA+ mRNA. Data are presented as ratio of each transcript measured in the two conditions. (**b**) 3'RACE of S- and AS-paRNAs in PC3 (top) and LNCaP (bottom) cells, respectively. Red bars indicate the positions of the transcripts 3'ends. Arrows indicate position of gene-specific primers used for 3'RACE. (**c**) S-paRNA level after knockdown of Rrp44 in PC3 cells. (**d**) 5'RACE of S- and AS-paRNAs in PC3 (top) and LNCaP (bottom) cells. Red bars indicate the positions of the transcripts 5'ends. Arrows indicate position of gene-specific primers used for 5'RACE. (**e**) paRNA detection by strand-specific RT-PCR with or without RNase treatment. Left, PC3 and LNCaP cells were transfected with pGL3 basic and S-TSS or AS-TSS reporters (pGL3-TSS). Right, PC3 cells were transfected with full length CDH1 promoter reporter. (**f**) Detection of S and AS transcripts in PC3 and LNCaP cells transfected with pGL3-basic, wild type (WT) or 5'deleted (Del) promoter reporter (left panel) and analysed by strand-specific RT-PCR (right panel).

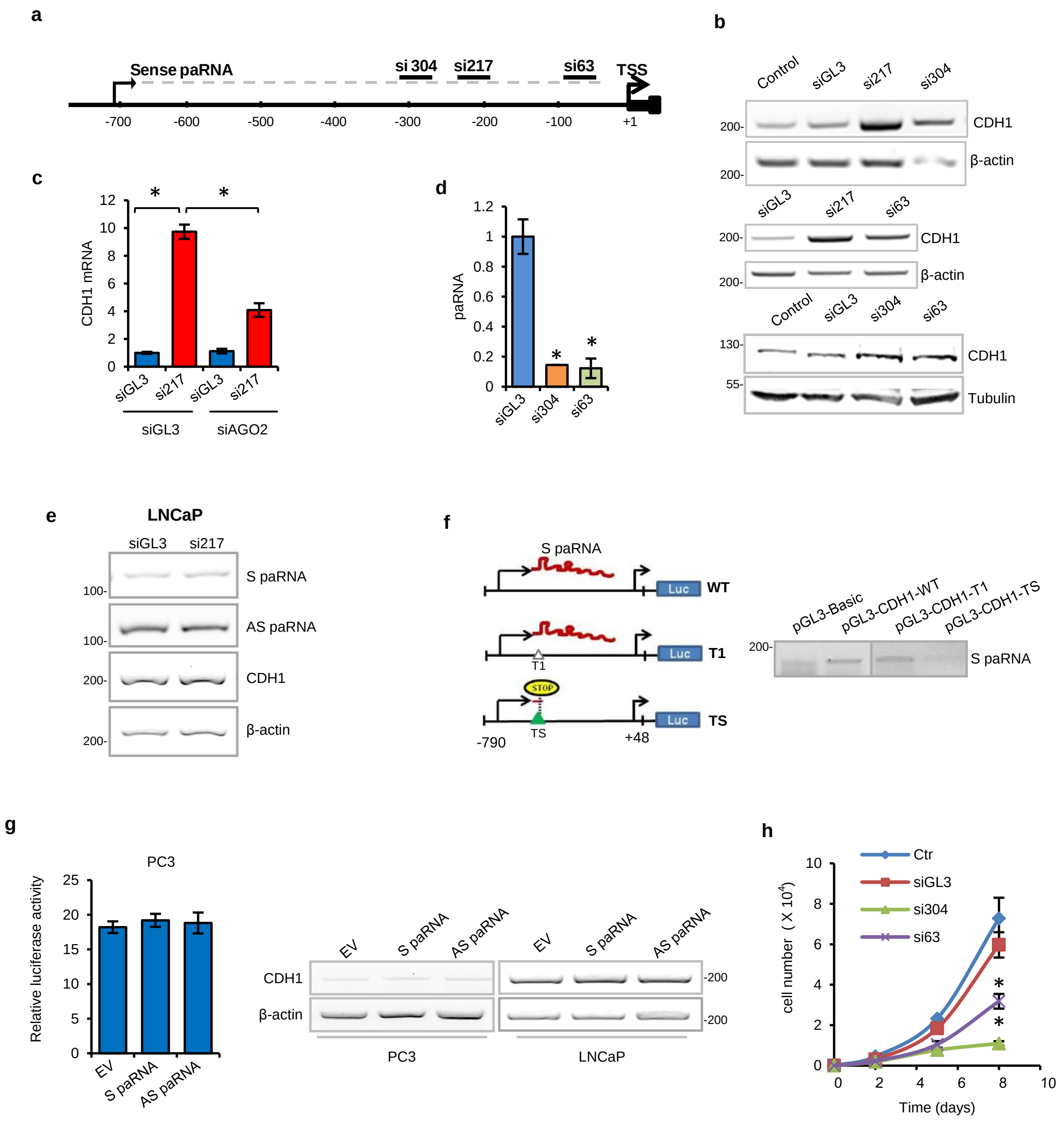

Supplementary Figure 3. siRNA-mediated targeting of promoter-associated RNAs at the CDH1 Gene. (**a**) Position of siRNA target sequences in the CDH1 promoter named according to their 5' nucleotide relative to the transcription start site (TSS; +1). (**b**) CDH1 mRNA in PC3 cells 72 h after transfection with si217 and si304 (top) and si217 and si63 (middle). E-cadherin protein levels in PC3 cells 72 h after transfection with si304 and si63 (bottom). (**c**) CDH1 mRNA in PC3 cells transfected with siAGO2 or siGL3 followed by si217 or siGL3. (**d**) paRNA levels in PC3 cells 24 h after transfection with siGL3, si304 and si63. (**e**) CDH1 mRNA and paRNA levels in LNCaP cells transfected with si217 and siGL3. (**f**) Detection of S-paRNA in PC3 cells transfected with pGL3-basic and CDH1 promoter reporter either wild type (WT) or with the insertion of a restriction site (T1) or a termination site (TS, SV40 polyA cassette) by strand-specific RT-PCR (right panel). Left panel, schematics of CDH1 promoter reporter constructs. (**g**) Activity of CDH1 promoter in luciferase reporter assay (left panel) and CDH1 mRNA level (right panel) in cells transfected with control vector (EV), sense (S-paRNA) or antisense (AS-paRNA) expression vector. Full length wild type CDH1 promoter reporter was co-transfected for the reporter assay. (**h**) Proliferation of PC3 cells transfected with si63, si304 and siGL3. Ctr, non-transfected control cells. Data are mean ± SD of three independent determinations. Student's t test was used for P value assessment. * P <0.05.

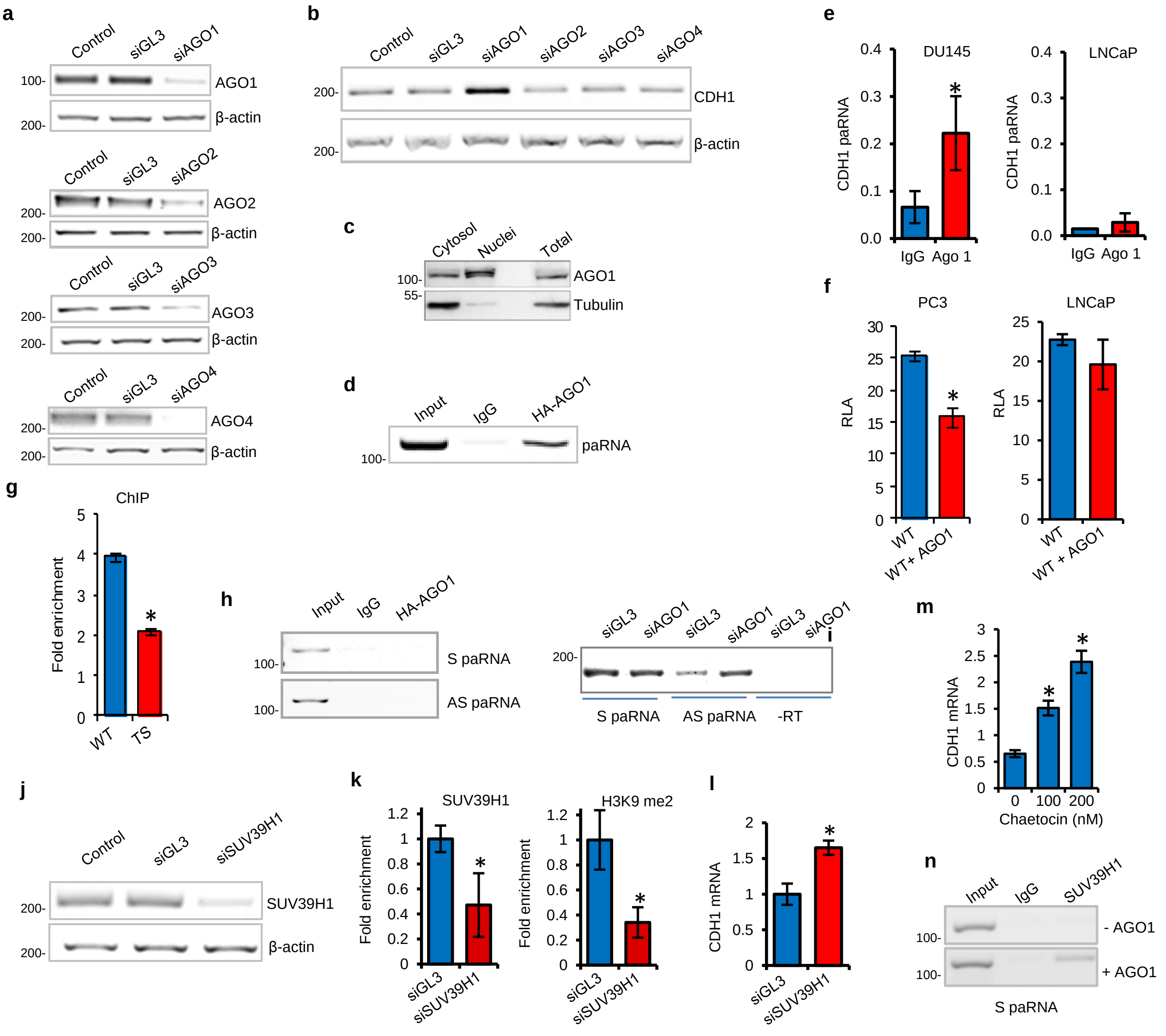

Supplementary Figure 4. Argonaute 1 is required for transcriptional regulation of CDH1. (**a**) Knockdown efficiency of Argonaute 1, 2, 3 and 4 in PC3 cells. Control, non-transfected cells. (**b**) CDH1 mRNA in PC3 cells transfected with siRNA targeting Argonaute 1, 2, 3 and 4. (**c**) Nuclear and cytoplasmic localization of AGO1 in PC3 cells. (**d**) Binding of HA-tagged Argonaute 1 (HA-AGO1) to promoter-associated RNA in PC3 cells by RIP. (**e**) Binding of AGO1to paRNA in DU145 and LNCaP cells by RNA-ChIP. (**f**) CDH1 promoter reporter activity in PC3 and LNCaP cells transfected with wild type (WT) alone or with HA-AGO1 (WT+AGO1). (**g**) Binding of HA-AGO1 to wild type (WT) CDH1 promoter reporter or mutated with termination site (TS) insertion. (**h**) AGO1 binding to S- and AS-paRNA in LNCaP cells transfected with the respective expression vectors. (**i**) Detection of S- and AS- paRNA by strand-specific RT-PCR in PC3 cells transfected with siGL3 or siAGO1. -RT, control reaction without RT step. (**j**) Efficiency of SUV39H1 knockdown by siRNA in PC3 cells. (**k**) SUV39H1 occupancy and H3K9me2 at the CDH1 promoter in PC3 cells transfected with siSUV39H1 or siGL3 and harvested after 72 h. (**l**) CDH1 mRNA in PC3 cells transfected with siSUV39H1. (**m**) CDH1 mRNA in PC3 cells treated with chaetocin. (**n**) Binding of SUV39H1 to promoter-associated RNA in PC3 cells transfected with S paRNA expression vector with or without HA-AGO1 vector. Data are mean ± SD of three independent determinations. Student's t test was used for P value assessment. * P <0.05.

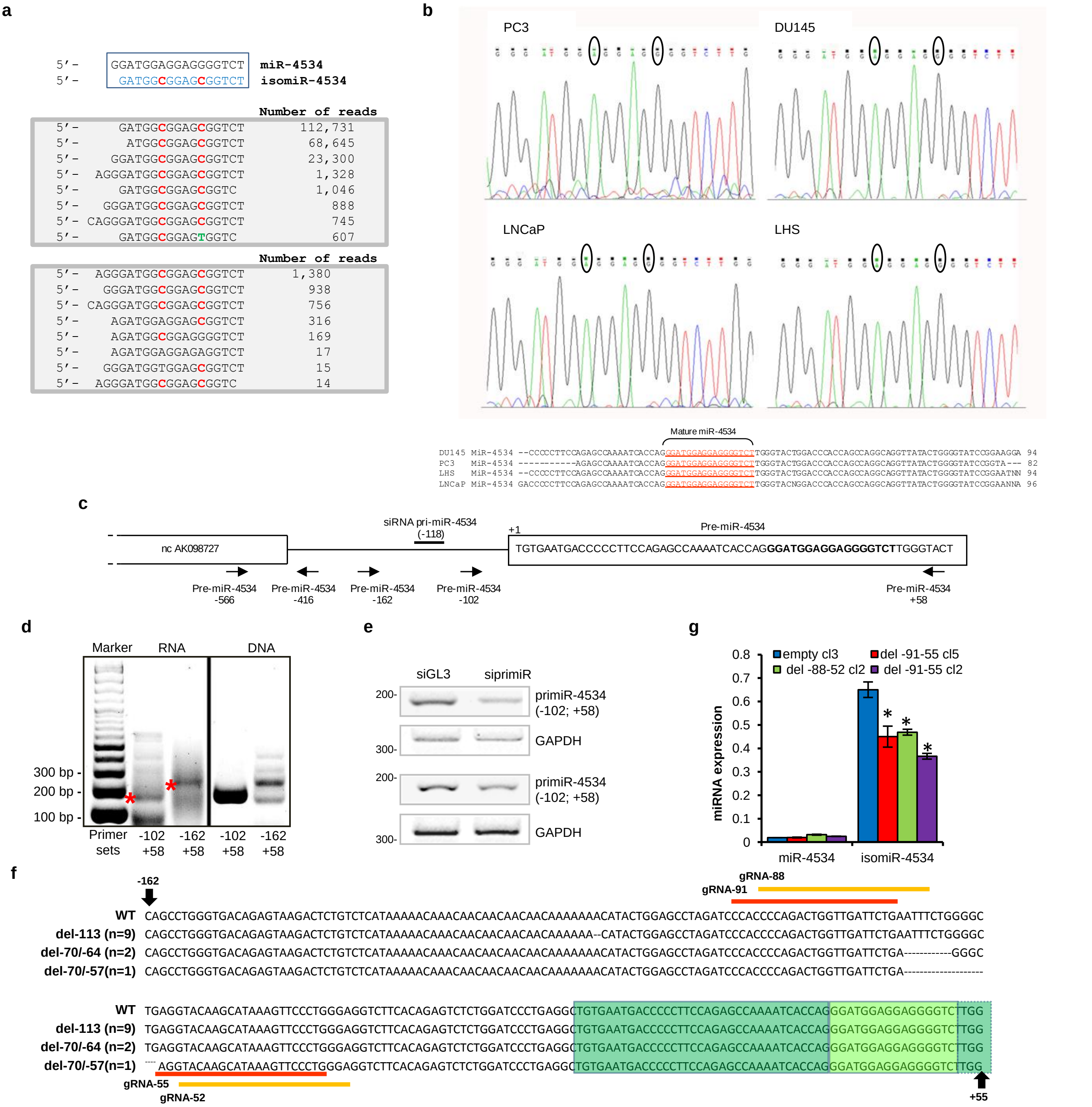

Supplementary Figure 5. Origin of isomiR-4534. (**a**) Alignment of miR-4534, isomiR-4534 and sequences retrieved from miRGator (top) and YM500 (bottom) small RNA-seq databases. (**b**) Genomic sequencing of the miR-4534 locus and sequence alignment in prostate epithelial cell lines. Circles, base changes in isomiR-4534. (**c**) Schematic representation of the pre-miR-4534 locus on chromosome 22 with position of PCR primer sets and siRNA target site. (**d**) Pri-miR-4534 transcript assessed by RT-PCR with the indicated primers sets. Genomic DNA (right panel) is shown as positive control of primer efficiency and amplicon size. (**e**) Pri-miR-4534 level in PC3 (top) and DU145 (bottom) cells transfected with siGL3 or pri-miR targeting siRNA (sipri-miR-4534). (**f**) Map of pre-miR-4534 locus and alignment of WT and targeted deleted sequences (delA -113 clones, n=9, and delATTTCTG -70-64 clones, n=2). Red circles indicate positions of deletions. Red and orange lines indicate the positions of the gRNA pair encoded by the two (del91-55 and del88-52) targeting constructs, respectively. Dark and light green boxes represent pre-miR-4534 and mature miR-4534 sequence, respectively. (**g**) miR-4534 and isomi-R453 levels in three targeted clones of DU145 cells obtained by transfection with del91-55 and del88-52 constructs and a control clones (empty vector). Data are mean ± SD of three independent determinations. Student's t test was used for P value assessment. * P <0.05.

a

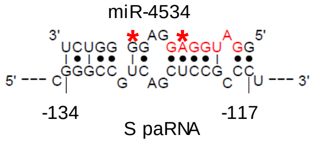

b

Input	Target database	Input sequence	Number of sites	Conserved sites	Number of target genes
hsa-miR-4534	Target Scan	GAUGGAG (nt 2-8; seed region)	157	157	155
hsa-isomiR-4534	Target Scan	AUGGCGG (nt 2-8; seed region)	7	7	7
hsa-isomiR-4534	Target Scan	GGCGGAG (nt 4-10; alt seed region)	9	9	9
hsa-miR-4534	DIANA MicroT v3	GGAUGGAGGAGGGGUCU (full miRNA sequence)	405	132	83
hsa-isomiR-4534	DIANA MicroT v3	GAUGGCGGAGCGGUCU (full miRNA sequence)	7	6	2

c

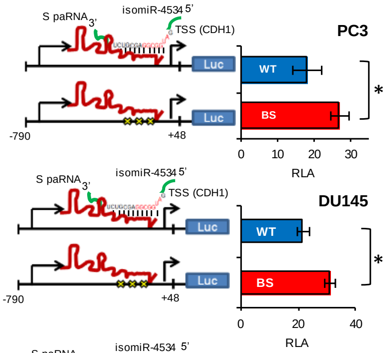

d

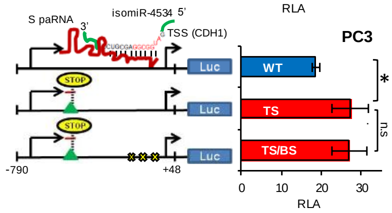

Supplementary Figure 6. IsomiR-4534 selective targeting of S-paRNA. (**a**) Predicted base pairing of miR-4534 with the isomiR-4534 target sequence in S-paRNA. Asterisks mark edited bases in isomiR-4534. (**b**) Number of predicted targets of miR-4534 and isomiR-4534 in 3' UTRs of human mRNAs retrieved using TargetScan and DIANA MicroTv3, taking into account seed regions or full length sequences of the miRNAs. (**c**) Luciferase reporter activity of wild-type (WT) and mutated (BS) promoter with altered isomiR-4534 binding site in PC3 (top) and DU145 (bottom) cells. (**d**) Luciferase reporter activity of wild-type (WT) and the reporter construct (TS) with termination site insertion and with or without the BS mutation tested in PC3 cells. Data are mean ± SD of three independent determinations. Student's t test was used for P value assessment. * P <0.05.

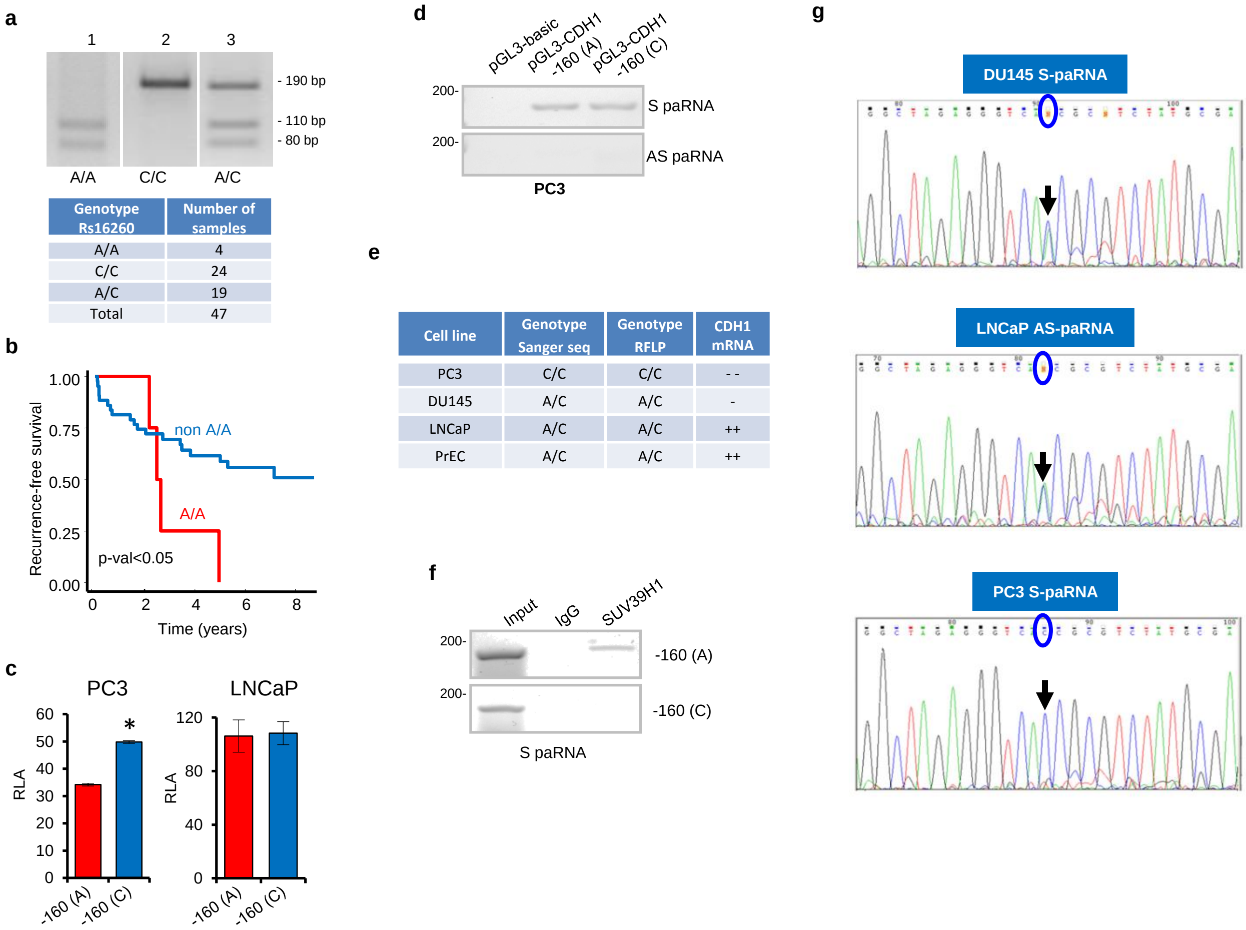

Genotype Rs16260	Number of samples
A/A	4
C/C	24
A/C	19
Total	47

Cell line	Genotype Sanger seq	Genotype RFLP	CDH1 mRNA
PC3	C/C	C/C	- -
DU145	A/C	A/C	-
LNCaP	A/C	A/C	++
PrEC	A/C	A/C	++

Supplementary Figure 7. SNP rs16260 in the CDH1 promoter. (**a**) SNP rs16260 genotype in human prostate tumors determined by RLFP analysis. Top, representative gel image. Bottom, genotype distribution in human tumors (*n=47*). (**b**) Relapse-free survival of prostate cancer patients (*n= 47*) grouped according to rs16260 genotype. (**c**) Promoter activity of the -160A and -160C promoter reporters in PC3 and LNCaP cells. (**d**) Synthesis of S and AS transcripts in PC3 cells transfected with the -160A or -160C CDH1 promoter reporters. (**e**) SNP rs16260 genotype in human prostate cancer cell lines determined by RFLP analysis and Sanger sequencing and comparative levels of CDH1 expression. (**f**) Allele-specific binding of SUV39H1 to S-paRNA in the presence of AGO1. (**g**) Rs16260 allele representation in S and AS promoter-associated RNAs in heterozygous (C/A) DU145 and LNCaP cells and homozygous (C/C) PC3 cells. S and AS transcripts were amplified by directional RT-PCR and the resulting amplicon sequenced. Circles indicate the position of the polymorphic site.

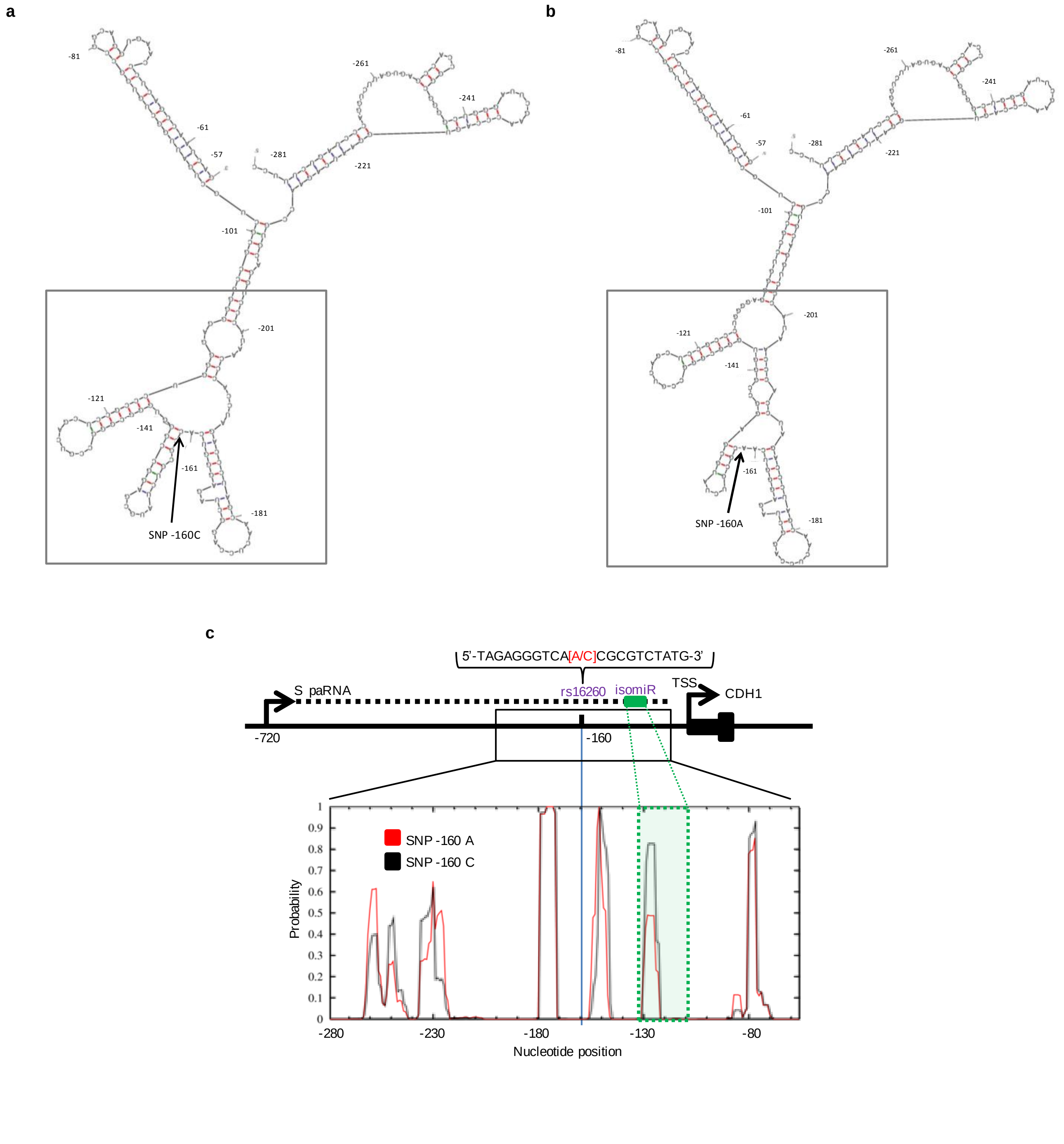

Supplementary Figure 8. Differential folding of sense promoter-associated RNA polymorphic variants. (**a**,**b**) *In silico* predicted folding of the S-paRNA (-281/-57) carrying either the -160 C (**a**) or -160A (**b**) alleles using Mfold. (**c**) Hairpin probability profiles of the S-paRNA region encompassing SNP rs16260 and isomiR-4534 binding sequence for the -160A and -160C allelic variants using Sfold.

a

SNP -160A

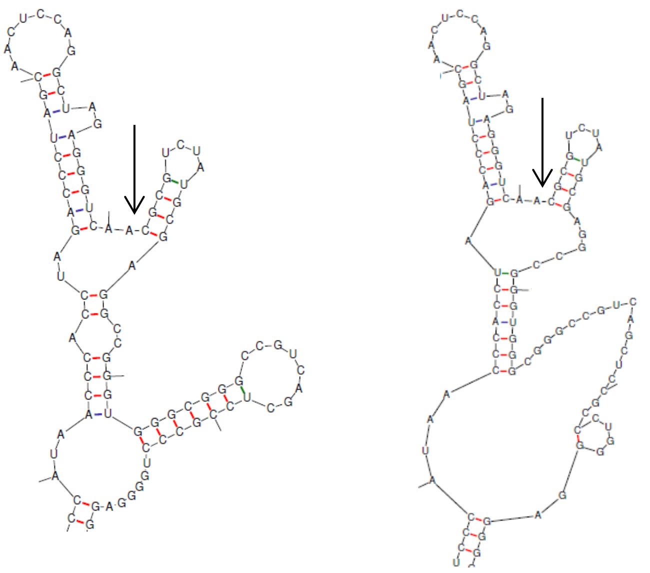

Non-forced single-strand structure

Forced single-strand structure

b

SNP -160C

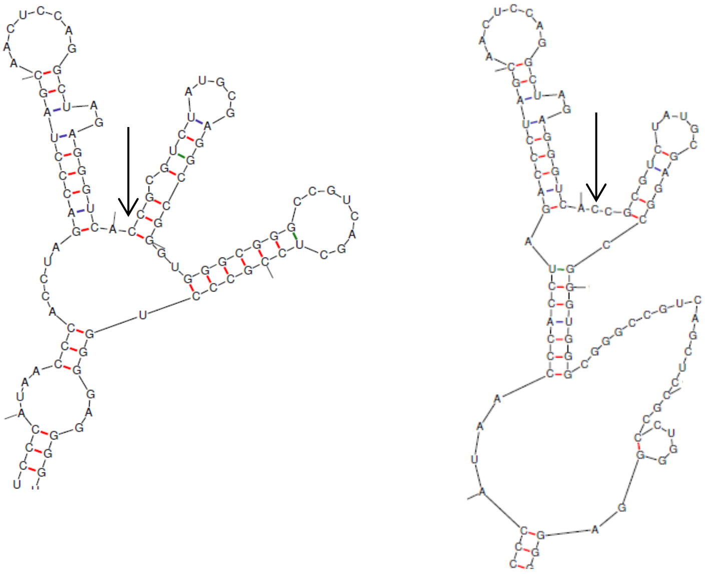

Non-forced single-strand structure

Forced single-strand structure

c

SNP	Non-forced ΔG (kcal/mol)	Forced ΔG (kcal/mol)	ΔΔG (kcal/mol)
160A	-87.44	-78.19	-9.21
160C	-92.94	-79.61	-13.33

Supplementary Figure 9. Differential unwinding of sense promoter-associated RNA polymorphic variants. (**a,b**) Predicted structures of the isomiR-4534 binding region in -160 A (**a**) or -160C (**b**) S-paRNA allelic variants generated using Mfold with (right) or without (left) forcing the hairpin sequence (-138 to -117) in single-strand conformation. (**c**) Estimated differences in Gibbs free energy between the two allelic variants.

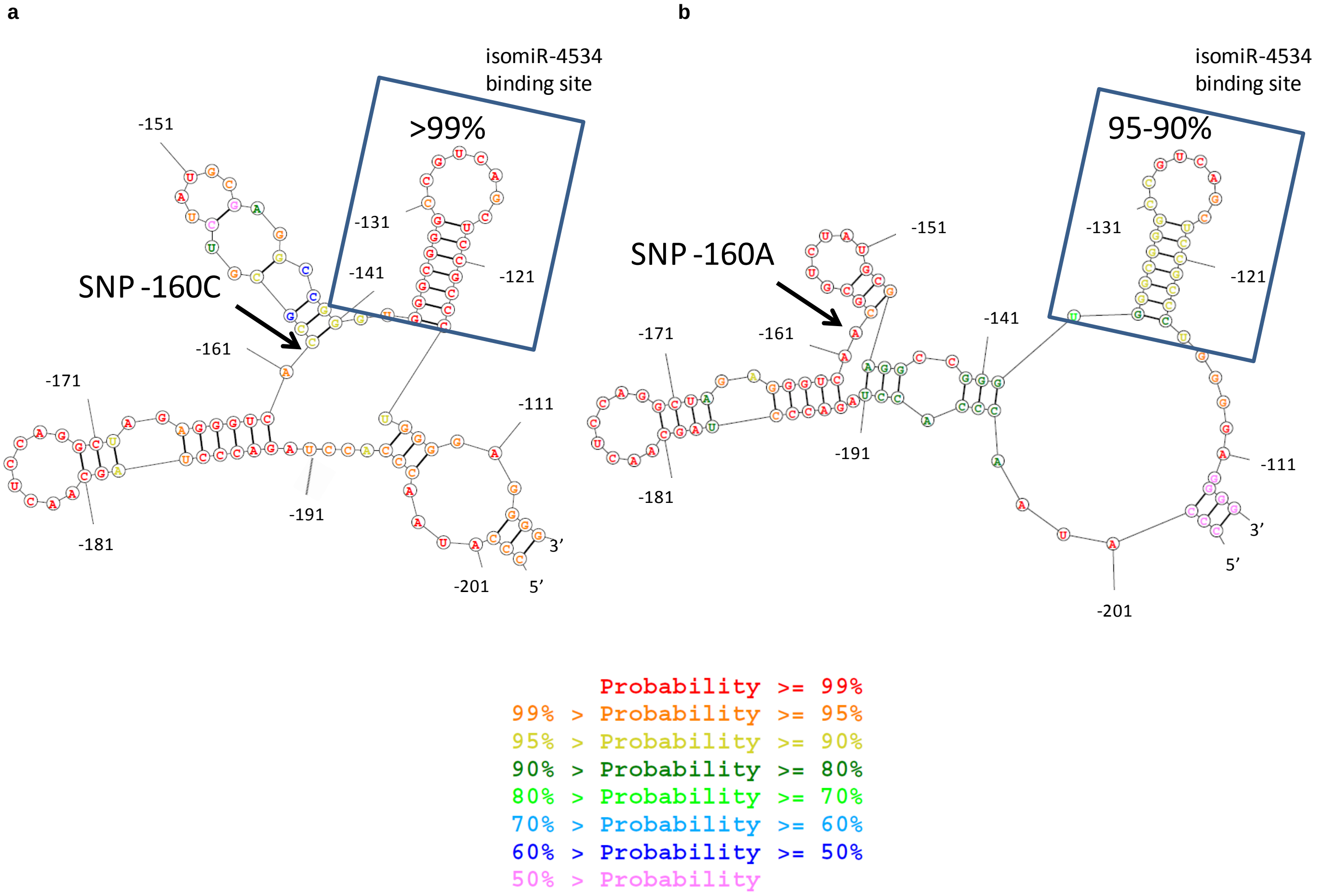

Supplementary Figure 10. Base pairing probability in the isomiR-4534 binding hairpin of sense promoter-associated RNA. (**a,b**) Estimates of pairing probability in the SHAPE based structures of the -160C (**a**) or -160A (**b**) S-paRNA allelic variants. Colour code indicates base pairing probability ranges.

a b

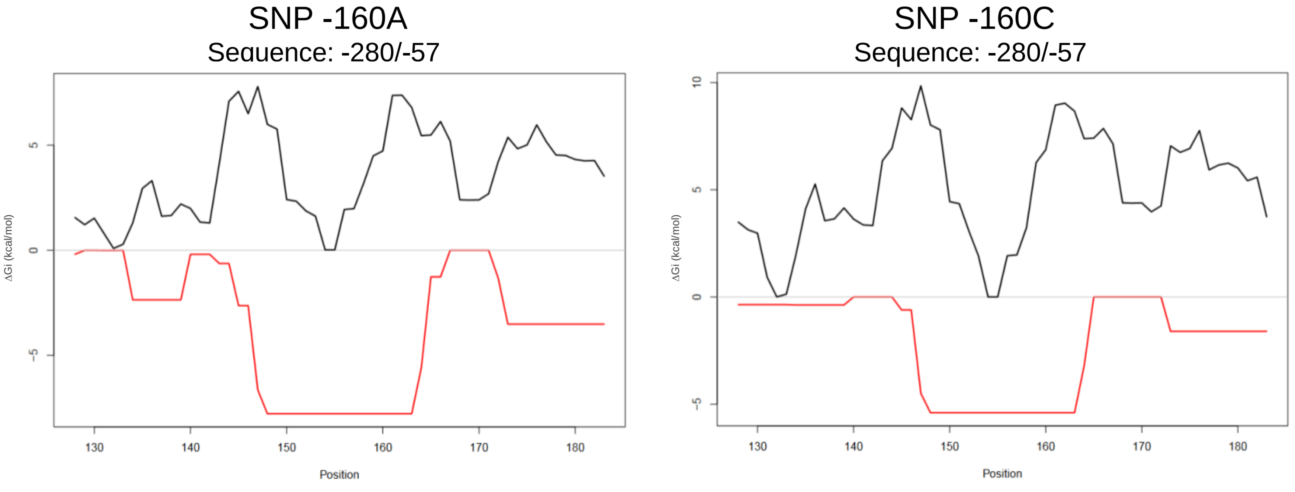

c

SNP	Opening energy ΔG (kcal/mol)	Free energy of binding ΔG (kcal/mol)
-160A	9.64	-7.78
-160C	12.02	-5.40

Supplementary Figure 11. Accessibility and binding capability of sense promoter-associated RNA to isomiR-4534. (**a,b**) Unwinding and RNA:RNA interactions examined for -160A (**a**) and -160C (**b**) S-paRNA allelic variants binding to isomiR-4534 using RNAup (Vienna Web package). Plots show unwinding energy for opening existing structures (black line) and interaction free energy (red line) for the two allelic variants. (**c**) Estimated unwinding energy and total binding energy for the two allelic variants.

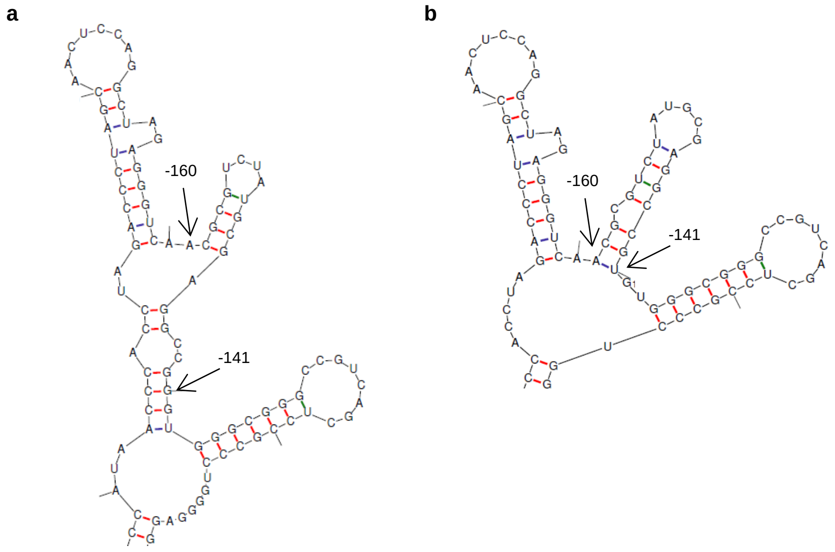

Supplementary Figure 12. Modeling the effect of single base mutation on the secondary structure of the AGO1/isomiR-4534 binding region. Predicted folding of the -160A (**a**) S-paRNA and the -160A/G141U (**b**) S-paRNA using Mfold.

Fig. 1d

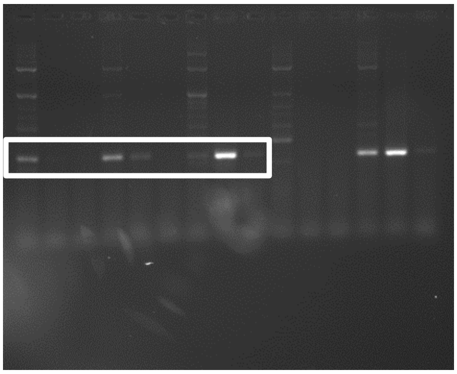

Fig. 1g

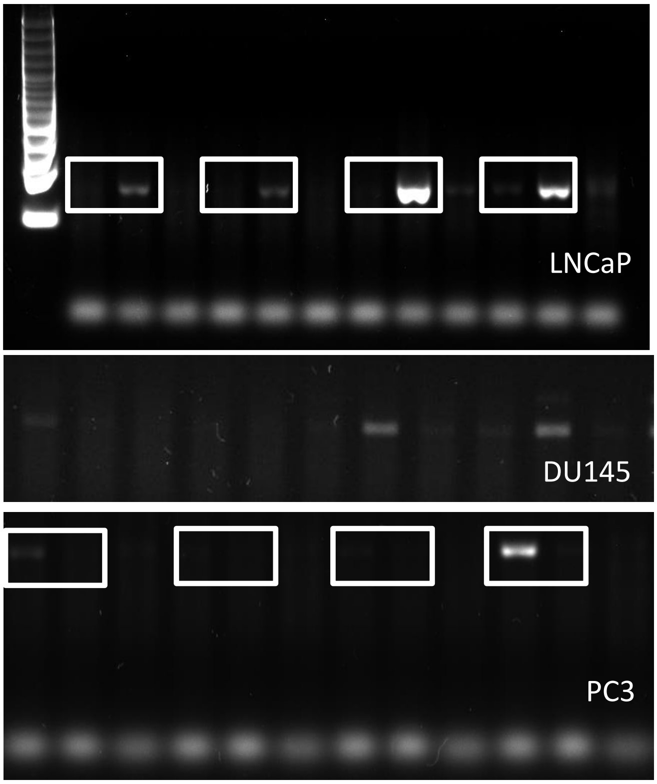

Fig. 1e

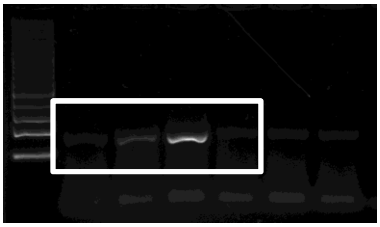

Fig. 1i

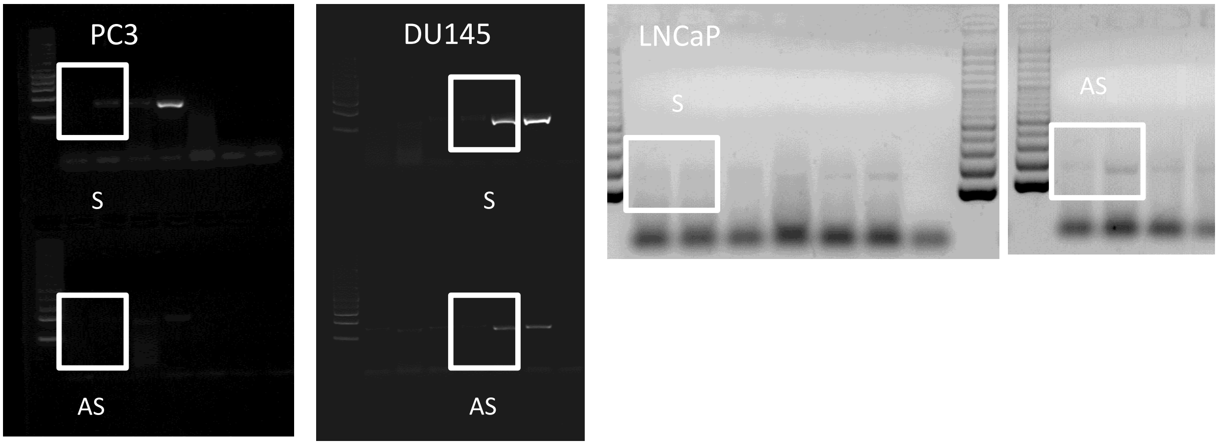

Fig. 1k

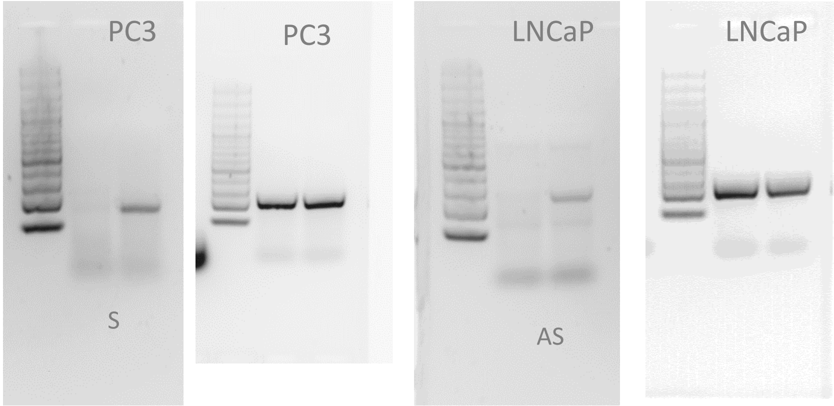

Supplementary Figure 13. Uncropped images related to Figure 1, panels 1d, 1e, 1g, 1i and 1k

Fig. 2a

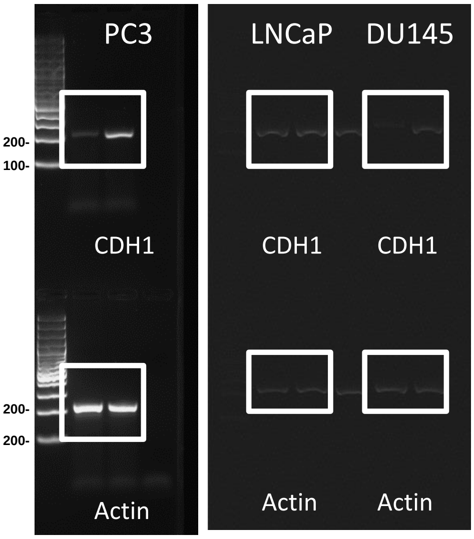

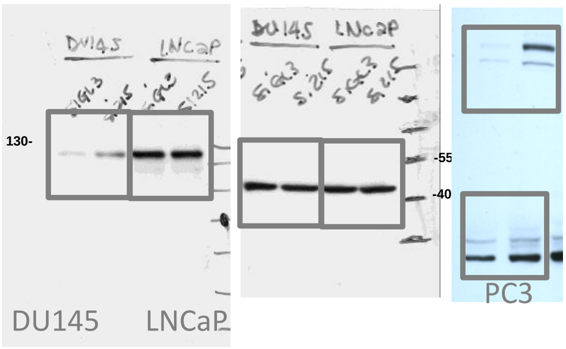

Fig. 3a

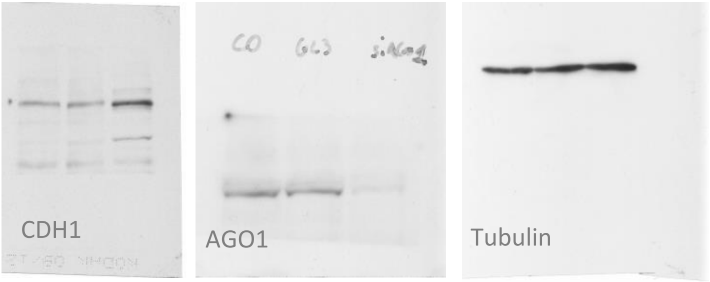

Fig. 3b

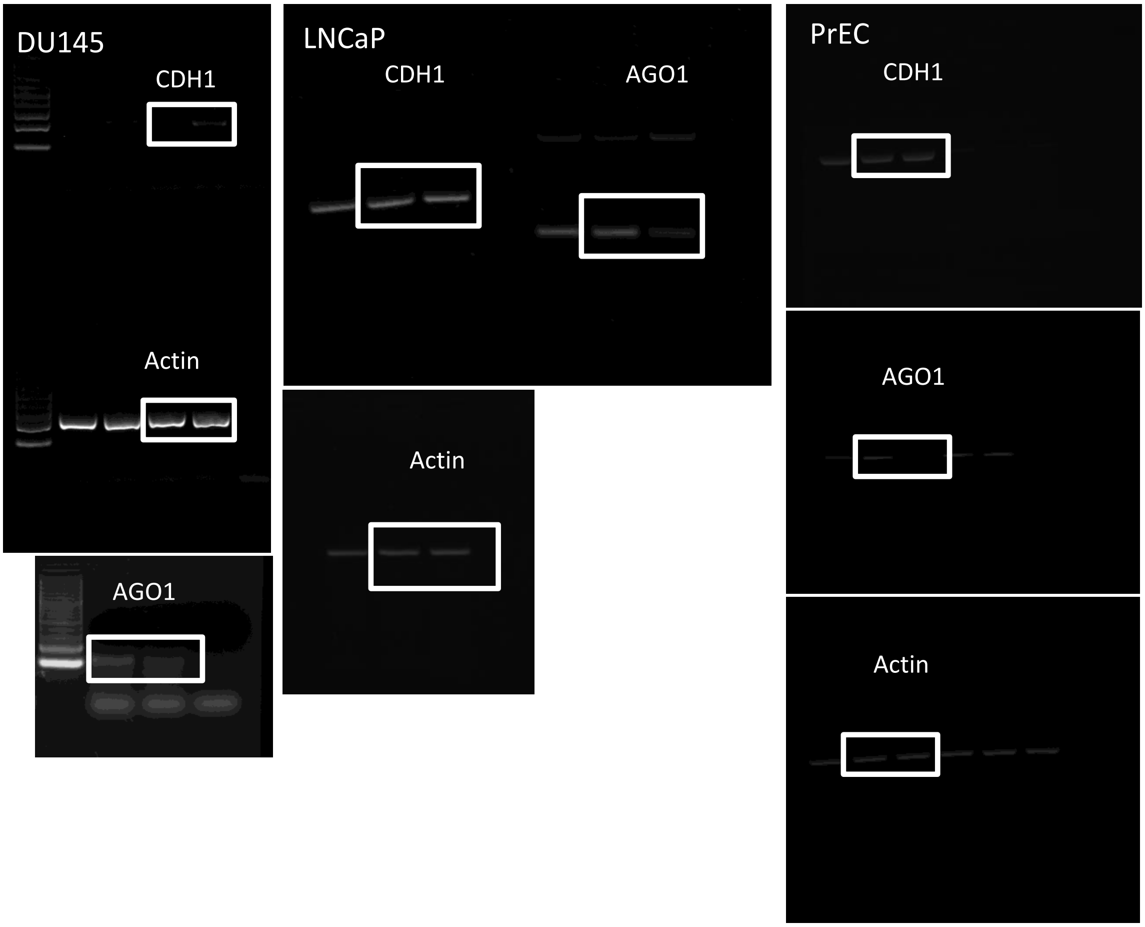

Supplementary Figure 14. Uncropped images related to Figure 2 and 3, panels 2a, 3a and 3b

Fig. 3e

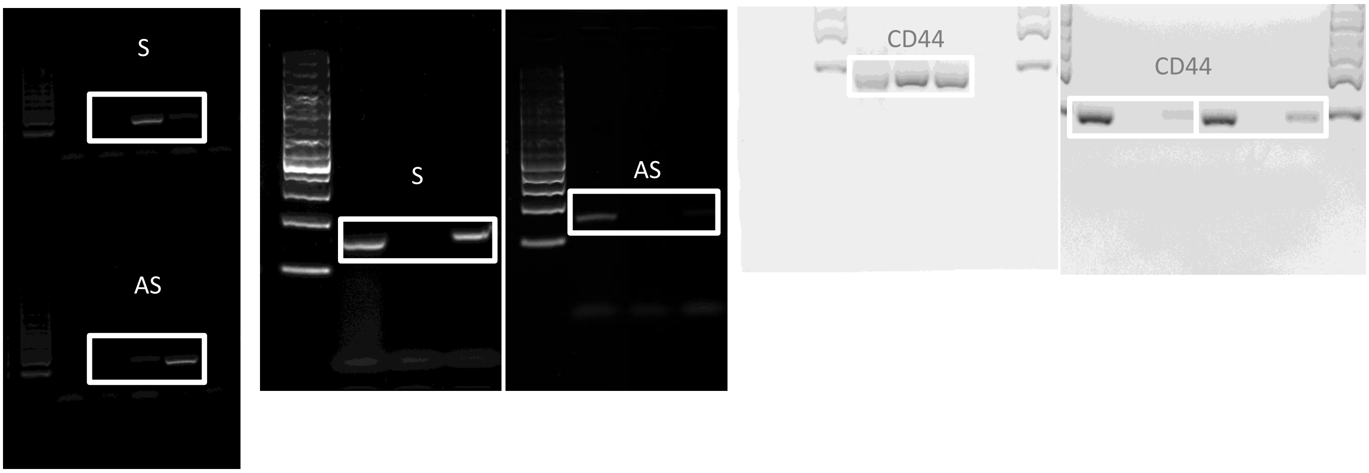

Fig. 3f

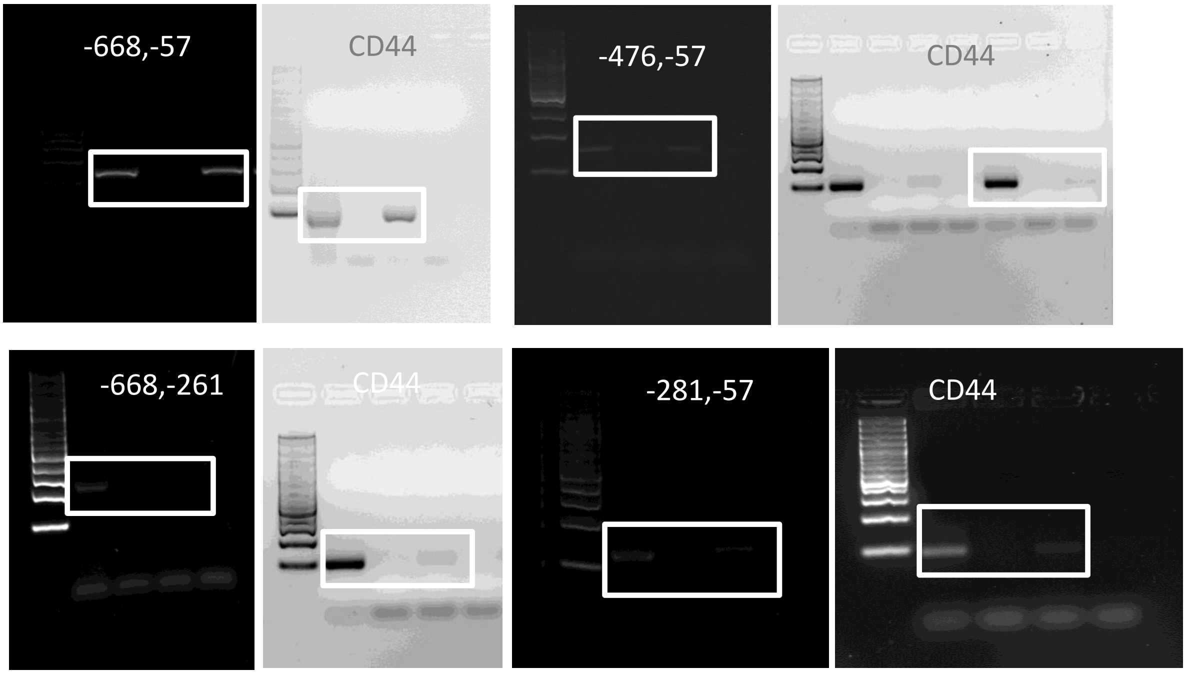

Supplementary Figure 15. Uncropped images related to Figure 3, panels 3e and 3f

Fig. 4i

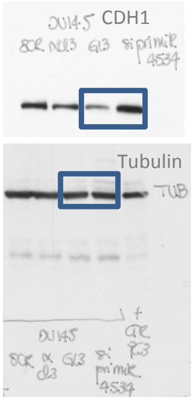

Fig. 5e

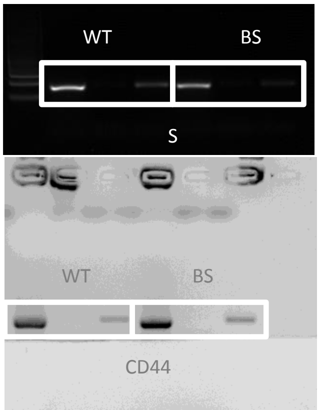

Fig. 6b

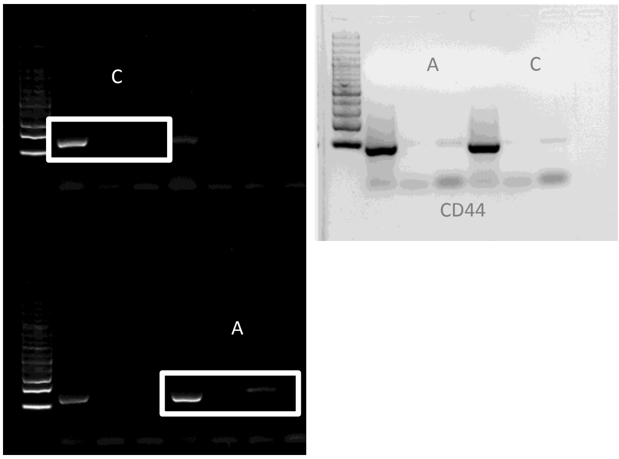

Fig. 7d

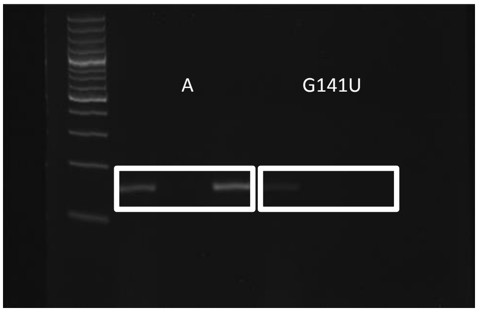

Supplementary Figure 16. Uncropped images related to Figure 4, 5, 6 and 7

Fig. S2c

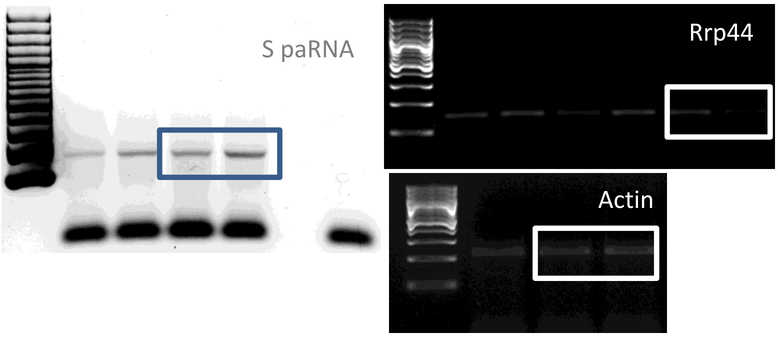

Fig. S2f

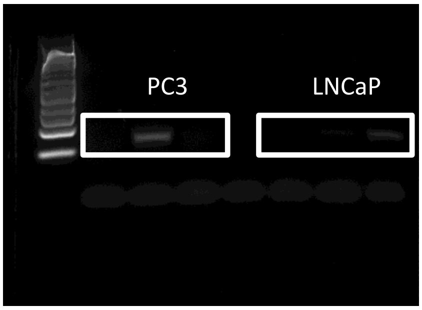

Fig. S3g

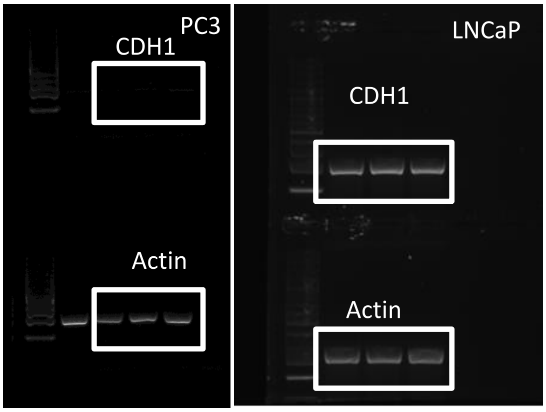

Fig. S4b

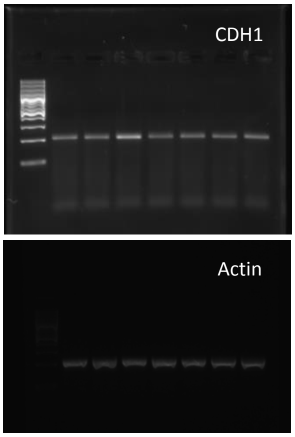

Fig. S4h

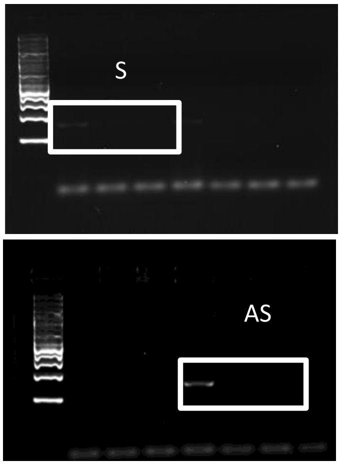

Fig. S4n

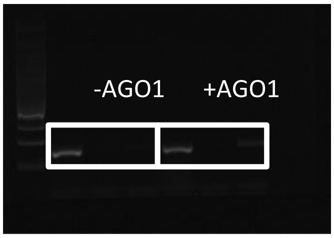

Fig. S7d

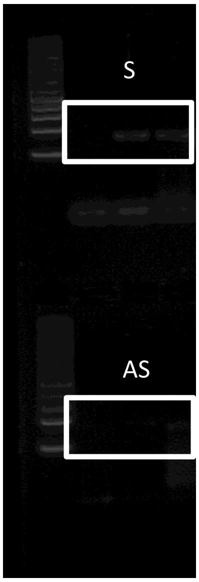

Fig. S7f

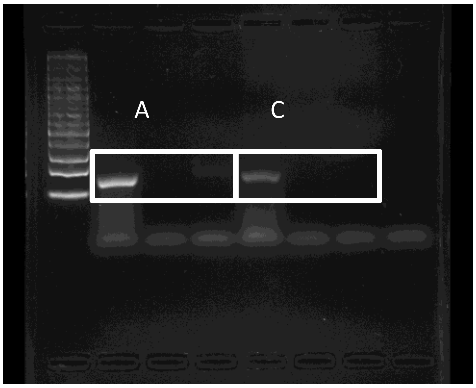

Supplementary Figure 17. Uncropped images related to Figure S2, S3, S4 and S7

Supplementary Table 1. Sequences of small interfering RNAs

siRNA ID	Sequence of forward and reverse strand
CDH1 si217	5’ AACCGUGCAGGUCCCAUAA 3’<br>5’ UUAUGGGACCUGCACGGUU 3’
CDH1 si63	5’ AAUCAGCGGUACGGGGGGC 3’<br>5’ GCCCCCCGUACCGCUGAUU 3’
CDH1 si304	5’ AGAACUCAGCCAAGUGUAA 3’<br>5’ UUACACUUGGCUGAGUUCU 3’
siSUV39H1	5’ AGAACAGCUUCGUCAUGGA 3’<br>5’ UCCAUGACGAAGCUGUUCU 3’
siAGO1	5’ GAGAAGAGGUGCUCAAGAAUU 3’<br>5’ UUCUUGAGCACCUCUUCUCUU 3’
siAGO2	5’ GCACGGAAGUCCAUCUGAAUU 3’<br>5’ UUCAGAUGGACUUCCGUGCUU 3’
siAGO3	5’ GAAAUUAGCAGAUUGGUAAUU 3’<br>5’ UUACCAAUCUGCUAAUUUCUU 3’
siAGO4	5’ GGCCAGAACUAAUAGCAAUUU 3’<br>5’ AUUGCUAUUAGUUCUGGCCUU 3’
siGL3	5’ CUUACGCUGAGUACUUCGA 3’<br>5’ UCGAAGUACUCAGCGUAAG 3’
sipri-miR-4534	5’ GAGGUACAAGCAUAAAGUU 3’<br>5’ AACUUUAUGCUUGUACCUC 3’
siRrp44	5’ GAAAGAGACUGAAACAGAA 3’<br>5’ UUCUGUUUCAGUCUCUUUC 3’

Supplementary Table 2. PCR, mutagenesis and sequencing primer sets

Primer ID	Forward (F) and Reverse (R) primer sequence
**CDH1**	
CDH1 +144 R	5' CTGCGGCTCCAAGGGCCCA 3’
CDH1 +610 F	5' TGCCCAGAAAATGAAAAAGG 3’
CDH1 +810 R	5' GTGTATGTGGCAATGCGTTC 3’
CDH1 +2497 F	5’ ATGAGTGTCCCCCGGTATCT 3’
CDH1 +2619 R	5’ ACGAGCAGAGAATCATAAGGGGCG 3’
CDH1 -1919 F	5’ CCAACATGATGAAACCCTGTC 3’
CDH1 -1759 R	5’ CTGGAGTGCAATGGTGTGTT 3’
CDH1 -476 F	5’ TGGTGGTGTGCACCTGTACT 3’
CDH1 -303 F	5’ GAACTCAGCCAAGTGTAAAAGC 3’
CDH1 -261 R	5’ AAGACCTGGGATCAGAAAGG 3’
CDH1 -238 F	5’ ATTCGAACCCAGTGGAATCA 3’
CDH1 -282 F	5’ CCCTTTCTGATCCCAGGTCT 3’
CDH1 -281 F	5’ CCTTTCTGATCCCAGGTCTT 3’
CDH1 -272 F	5’ TCCCAGGTCTTAGTGAGCCA 3’
CDH1 -204 R	5’ GACCTGCACGGTTCTGATTC 3’
CDH1 -190 R	5’ TAGGTGGGTTATGGGACCTG 3’
CDH1 -171 R	5’ GCCTGGAGTTGCTAGGGTCT 3’
CDH1 -153 R	5’ AGACGCGGTGACCCTCTA 3’
CDH1 -139 R	5’ CACCCGGCCTCGCATAGA 3’
CDH1 -82 R	5’ GGCCACAGCCAATCAGCA 3’
CDH1 -57 R	5’ CTGATTGGCTGAGGGTTCAC 3’
CDH1 -180_C F	5’ ACTCCAGGCTAGAGGGTTAC 3’
CDH1 -180_A F	5’ ACTCCAGGCTAGAGGGTTAA 3’
CDH1 EX3 F	5’ CTCGACACCCGATTCAAAGT 3’
CDH1 EX3 R	5’ GGTGGTGCCCCACTGTATT 3’
CDH1 EX8 F	5’ CGTATACCCTGGTGGTTCAAG 3’
CDH1 EX8 R	5’ GGAGGATTATCGTTGGTGTCA 3’

**ACTIN**	
ACT +221 F	5' AAGAGAGGCATCCTCACCCT 3'
ACT +439 R	5' TACATGGCTGGGGTGTTGAA 3'
ACT +778 F	5' ATTGGCAATGAGCGGTTC 3'
ACT +864 R	5' GGATGCCACAGGACTCCAT 3'
**CD44**	
CD44 F	5' GACACCATGGACAAGTTTTGG 3'
CD44 R	5' CGGCAGGTTATATTCAAATCG 3'
**Rrp44**	
Rrp44 F	5' GTGGCATGCTTTCCAAGTCT 3'
Rrp44 R	5' GCCAACCATCAATAGCAACA 3'
**ARGONAUTE 1**	
AGO1 F	5' GCACTGCCCATTGGCAACGAA 3'
AGO1 R	5' CATTCGCCAGCTCACAATGGCT 3'
**ARGONAUTE 2**	
AGO2 F	5'CGCGTCCGAAGGCTGCTCTA 3'
AGO2 R	5'TGGCTGTGCCTTGTAAAACGCT 3'
**ARGONAUTE 3**	
AGO3 F	5'GGAATTAGACAAGCCAATCAGCA 3'
AGO3 R	5'AGGGTGGTCATATCCTTCTGGA 3'
**ARGONAUTE 4**	
AGO4 F	5'CTAACAGACTCCCAGCGTGTCA 3'
AGO4 R	5'GACTGGCTGGCCGTCTAGTCA 3'
**SUV39H1**	
SUV39H1 F	5' GGCAACATCTCCCACTTTGT 3'
SUV39H1 R	5'CAATACGGACCCGCTTCTTA 3'
**Pri-miR-4534**	
Pri-miR-4534 -566 F	5'CCTAGATCCCACCCCAGACT 3'
Pri-miR-4534 -416 R	5'ACGCCTGGCTCAGTATGTTT 3'
Pri-miR-4534 -162 F	5'CAGCCTGGGTGACAGAGTAAG 3'

Pri-miR-4534 -102 F	5'CCTAGATCCCACCCCAGACT 3'
Pri-miR-4534 +58 R	5'TACCCAAGACCCCTCCT 3'
**Pre-miR-4534**	
Pre-miR-4534 -104 F	5'AAGTTCCCTGGGAGGTCTTC 3'
Pre-miR-4534 +99 R	5'TCCGGATACCCCAGTATAACC 3'
**Mutagenesis primers**	
CDH1-160SNP F	5'AGGCTAGAGGGTCACCGCGTCTATGCGAGG 3'
CDH1-160SNP R	5'CCTCGCATAGACGCGGTGACCCTCTAGCCT 3'
CDH1-PROMOTER-T1 F	5'CTGCTAGCTCAGTGGGCCCTGGCGAATTCCTGAAATCCTAGCAC 3'
CDH1-PROMOTER-T1 R	5'GTGCTAGGATTTCAGGAATTCGCCAGGGCCCACTGAGCTAGCAGCCT 3'
CDH1-PROMOTER-BS F	5'GGGCGGGCCGTCAGCACGGGCCTGGGGAGGGGTC 3'
CDH1-PROMOTER-BS R	5'GACCCCTCCCCAGGCCCGTGCTGACGGCCCGCCC 3'
**S_TSS**	
S_F	5' CAAACTAGCAAAATAGGCTGTCC 3'
S_R	5' AAGCTTCGGAGTCTCGCTCTGTCTTG 3'
**AS_TSS**	
AS_F	5' AAGCTTCCTTTCTGATCCCAGGTCTT 3'
AS_R	5' GAGCTCCTGATTGGCTGAGGGTTCAC 3'
**S-AS transcript chimera**	
S-AS_F1	5' AGACTCCGAAGCTTGGCAT 3'
S-AS_F2	5' TAGGTGGGTTATGGGACCTG 3'
S-AS_R	5' GGAACCAGGGCGTATCTCTT 3'
**SHAPE primers**	
pcDNA-120 – paRNA F	5' CCACTGCTTACTGGCTTATCG 3'
pcDNA-paRNA +61 polyA R	5' TTT TTT TTT TTT TTT CAACAGATGGCTGGCAACTA 3'
extREV9	5' CAACAGATGGCTGGCAACTA 3'
extREV8	5' TCTAGACTCGAGCGGCCGCCA 3'
extREV5_A	5' AGACGCGTTGACCCTCTA 3'
extREV5_C	5' AGACGCGGTGACCCTCTAGC 3'

Supplementary Table 3. Predicted paRNA in the CDH1 locus

paRNA	Cell line	Chrom	Start	End	Strand	HOMER annotation	Close gene	Score
AS	lncap	chr16	68770846	68771078	-	Promoter-antisense	CDH1	24
AS	hct p53 -/-	chr16	68770844	68771044	-	Promoter-antisense	CDH1	23
AS	hct p53 +/+	chr16	68770865	68771045	-	Promoter-antisense	CDH1	13
AS	mcf7 c1	chr16	68770810	68771042	-	Promoter-antisense	CDH1	18
AS	h1esc t1	chr16	68770852	68771039	-	Promoter-antisense	CDH1	11
S	hct p53 -/-	chr16	68770887	68771056	+	Promoter-sense	CDH1	22
S	hct p53 -/-	chr16	68770523	68770676	+	Not annotated for score filter	CDH1	0.4
S	hct p53 -/-	chr16	68770731	68770881	+	Not annotated for score filter	CDH1	0.666667
S	hct p53 +/+	chr16	68770512	68770729	+	Not annotated for score filter	CDH1	0.56

.
